# Measures and Metrics of Biological Signals

**DOI:** 10.3389/fphys.2018.01707

**Published:** 2018-12-04

**Authors:** Obrad Kasum, Aleksandar Perović, Aleksandar Jovanović

**Affiliations:** ^1^Group for Intelligent Systems (GIS), Faculty of Mathematics, University of Belgrade, Belgrade, Serbia; ^2^Faculty of Transport and Traffic Engineering, University of Belgrade, Belgrade, Serbia

**Keywords:** measures of biological signals, metrics on biological signals, complexity, dimension, similarity

## Abstract

The concept of biological signals is becoming broader. Some of the challenges are: searching for inner and structural characteristics; selecting appropriate modeling to enhance perceived properties in the signals; extracting the representative components, identifying their mathematical correspondents; and performing necessary transformations in order to obtain form for subtle analysis, comparisons, derived recognition, and classification. There is that unique moment when we correspond the adequate mathematical structures to the observed phenomena. It allows application of various mathematical constructs, transformations and reconstructions. Finally, comparisons and classifications of the newly observed phenomena often lead to enrichment of the existing models with some additional structurality. For a specialized context the modeling takes place in a suitable set of mathematical representations of the same kind, a set of models M, where the mentioned transformations take place. They are used for determination of structures **M**, where mathematical finalization processes are preformed. Normalized representations of the initial content are measured in order to determine the key invariants (characterizing characteristics). Then, comparisons are preformed for specialized or targeted purposes. The process converges to the measures and distance measurements in the space **M**. Thus, we are dealing with measure and metric spaces, gaining opportunities that have not been initially available. Obviously, the different aspects in the research or diagnostics will demand specific spaces. In our practice we faced a large variety of problems in analysis of biological signals with very rich palette of measures and metrics. Even when a unique phenomena are observed for slightly different aspects of their characteristics, the corresponding measurements differ, or are refinements of the initial structures. Certain criteria need to be fulfilled. Namely, characterization and semantic stability. The small changes in the structures have to induce the small changes in measures and metrics. We offer a collection of the models that we have been involved in, together with the problems we met and their solutions, with representative visualizations.

## Introduction

A biological signal is any mapping (change) of a biological quantity/content into the corresponding set (codomain), with the purpose to represent the particular process in a form suitable for studying, monitoring, determination of functional connections (relations, dependence) between the studied quantity and its relevant constituents.

The change in biological quantities can have particular significance and lead to discovering of deep processes hidden from the direct (e.g., visual) observations. Long ago, it was discovered that biological organisms function through a sequence of interconnected processes-results of action of systems and subsystems within a hierarchically organized functions.

Hence, it is prudent to formally define a biological signal as a function of the form *f*:*D* → *M* that maps the initial set of biological phenomena (states, conditions, etc.) *D* into the corresponding codomain *M* of measurement data. For example, such signals are the usual home or clinical measurement of the body temperature, arterial pressure or atmospheric pressure, performed sporadically or at regular intervals. The purpose is to determine their current values, and to monitor their change in time in order to identify the presence of pathology, or to control the side effects of introduced drugs that may have negative impact on systems regulating vital functions.

Certain biological phenomena such as body temperature or blood pressure are analog. The number of erythrocytes, or the number of bacteria per space unit are examples of digital signals, but with a large number of units. The corresponding measuring procedures are designed to obtain the suitable approximations within some finite scale. For example, the body temperature of a living human is scaled by the degrees in Celsius, with min = 35°*C* and max = 42°*C*. Similarly, the blood pressure is scaled by a height in millimeters of Hg in a capillary tube, while the number of erythrocytes is scaled in thousands per space unit etc. The basic concepts about analog and digital quantities can be found in textbooks (Attenborough, 2003; Werde and Spiegel, 2010; Hsu, 2014).

On the other hand, measurements are performed in order to assess the presence or the absence of a property/pathology. As such, they are composed with the additional binary scale (absence, presence), ternary scale (absence, presence, strong presence), and so on. Some phenomena require more complex structures involving indications of inner dependences, usually represented by multigraphs.

Regardless of the form of the performed measurements, the modern computers are at such technical level that allows implementation of various numerical and symbolic algorithms related to acquisition, representation, analysis and transformation/manipulation of biological signals. Hence, the modern representation of biological signals use mathematical structures (numerical or abstract) suitable for digitization, exact representation, deeper insights and finally, classification. In the very rich variety of biological signals, here we focus on some mathematical representation and operation aspects involving broad range of applications, thus illustrating the rich abundance of phenomena and their mathematical treatment, rather than trying to have more complete approach, which needs much larger space and more complex method coverage.

Automated acquisition and processing of biological signals has opened the possibility of elimination of subjectivity in validation and interpretation of a measurement. At the same time, digitization has enabled application of the large mathematical apparatus, making possible nontrivial transformations of the initial content. The large number of scientific breakthroughs that are made in this way has established the new, highly prominent scientific discipline involving broad mathematical modeling and their computer implementations.

Developing some systems for operation with biological signals, in our group GIS (Group for Intelligent Systems), we have implemented systems for digital upgrades of the existing analog research and clinical equipment for the measurement of e.g., arterial pressure, ECG, EEG, specific neurology, ultrasound, NMR, and digital microscopy signals. Those systems have enabled digital acquisition of the various types of related signals, including biometric parameters like voice and fingerprints, acquisition of various molecular biology signals like chromosomes and genetic sequences. We have also implemented tools for representation, visualization, manipulation and transformation of signals and integrated it with the CCD computerized microscopy.

In particular, developed software solutions include: signal monitoring, acquisition and real time analysis (the first version was implemented in 1994); image acquisition and analysis (1994); image spectroscopy (1995), photomorphology (1995–1998), color combine fluorescent microscopy (1997–1998); automatized karyotyping involving object recognition, normalization, and classification (1997).

As mentioned above, before implementation, all measurements and analyses were performed manually by direct observation. The improvement in efficiency and precision was immediately observed by the involved researches. Developed solutions have been in use for almost two decades at more than 20 research laboratories at the University of Belgrade, Lomonosov state university at Moscow, and UC Berkeley, see (Jovanović, 2001; Jovanović et al., 2014).

In addition, we have also developed hardware for those laboratories including CCD microscopes, computerized EEG, ECG, CTG, acoustic RT spectroscopes, equipment for recording of magnetic field attenuation etc. (see Jovanović, 2001; Jovanović et al., 2014).

**Figure 1:**
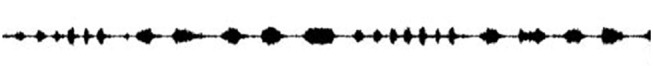
A mammal acoustic signal.

**Figure 2:**
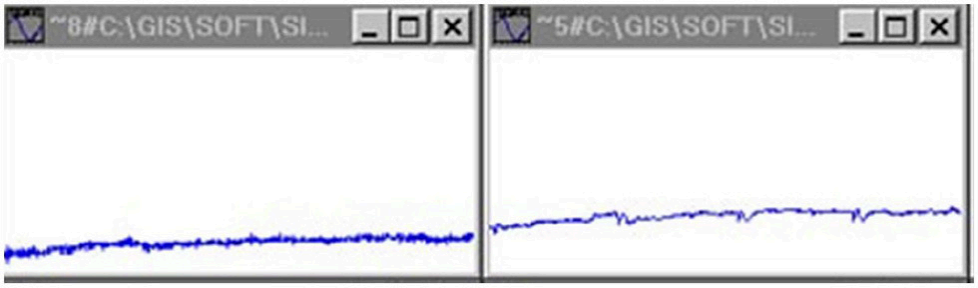
Arterial pressure signals with implanted transducers (rats) (Jovanović, 2001; Jovanović et al., 2014).

Those systems have enabled precise measurements, significant reduction of errors previously made by subjective visual detection of important features, nontrivial numerical, algebraic, geometrical, topological, and visual transformations of the acquired signals and integration with other related computerized systems. In particular, images displayed at Figures 1, 2, 4, 7–9, 12 and 21 were produced by the mentioned software for signal acquisition, processing and visualization.

**Figure 3:**
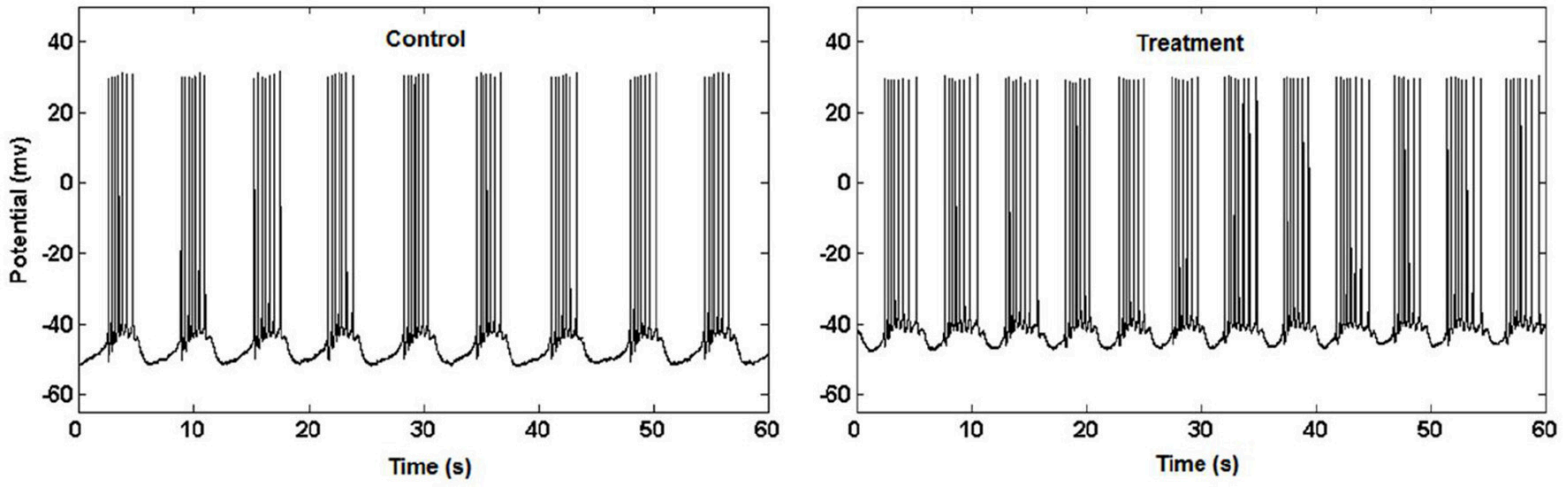
Garden snail neuron activity (Kesić et al., 2014).

**Figure 4:**
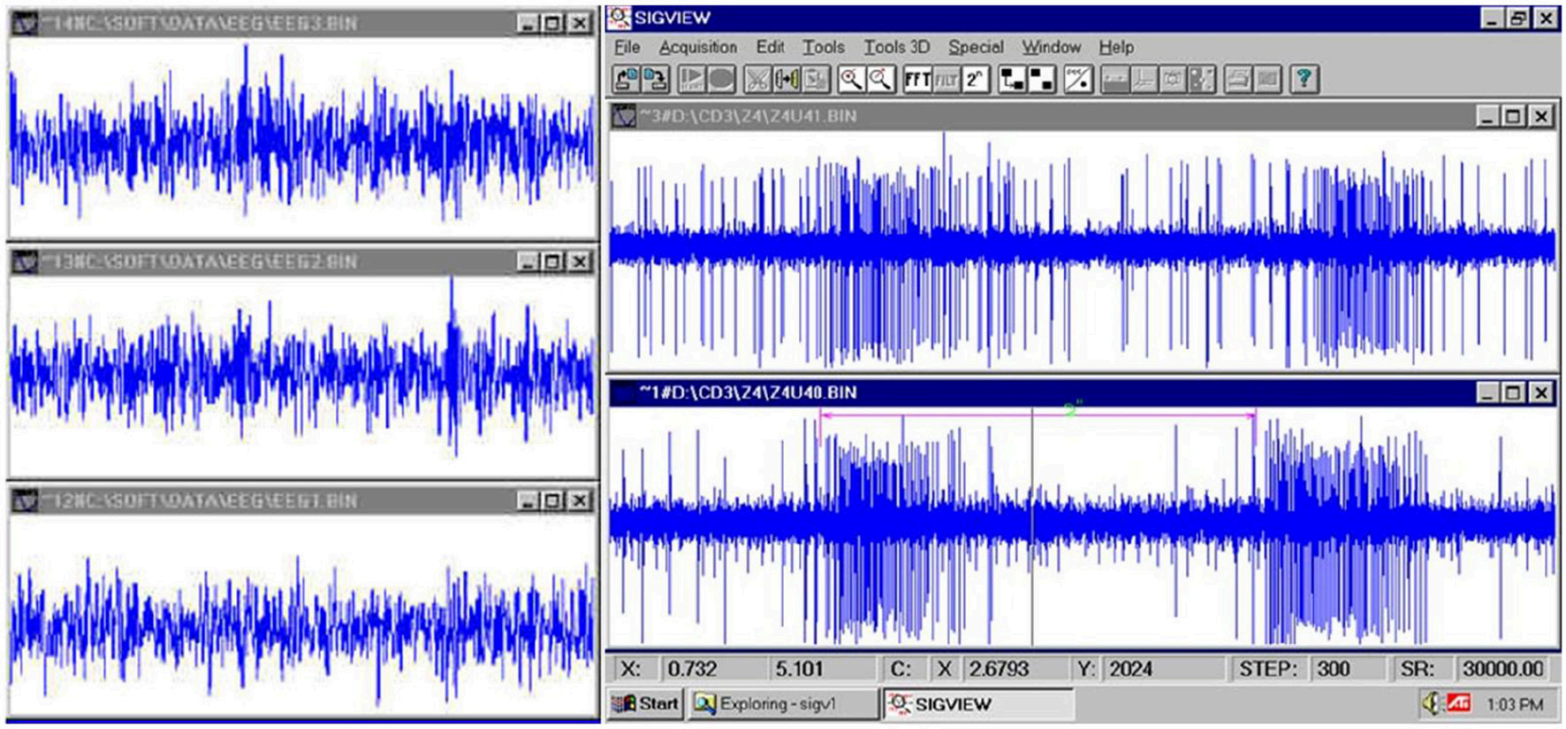
**(Left)**: fast EEG; **(Right)**: very fast firing of a neuron (Jovanović, 2001; Jovanović et al., 2014).

**Figure 5:**
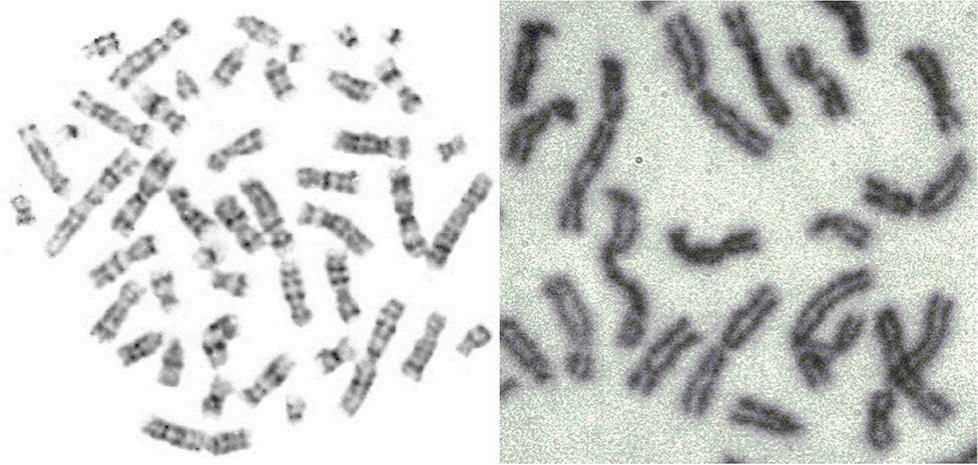
Mitosis-two chromosome distributions (Jovanović, 2001; Jovanović et al., 2014).

**Figure 6:**
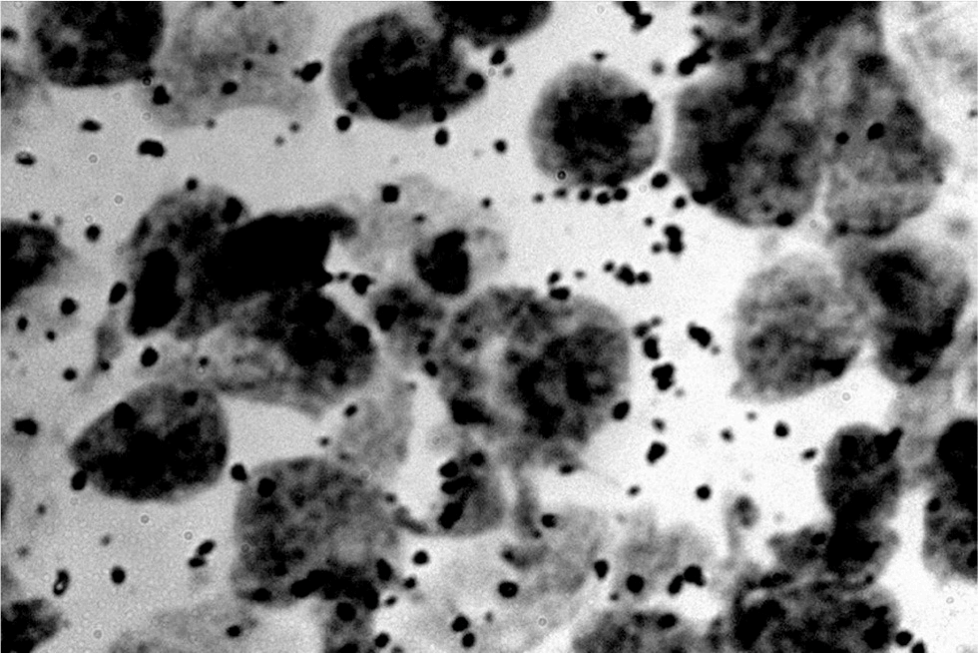
RNA dots related to the neuron nucleus (rats) (Jovanović, 2001; Jovanović et al., 2014).

**Figure 7:**
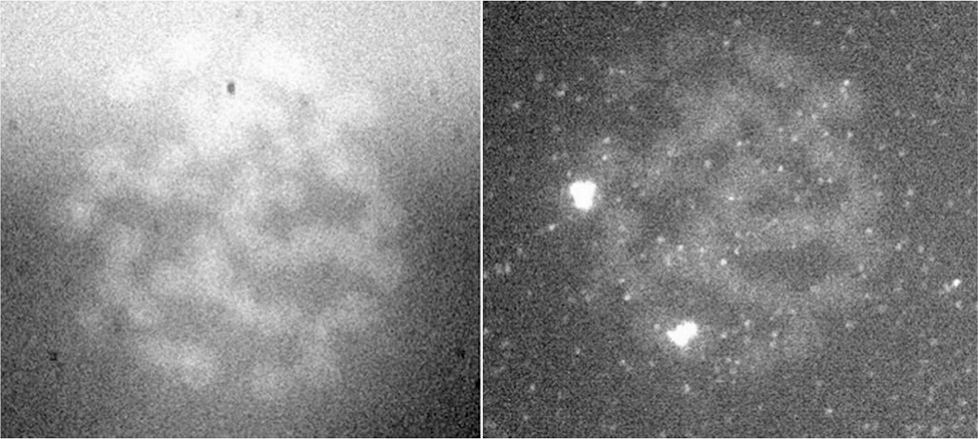
FISH signal of the same preparation in two different wavelengths (Jovanović, 2001; Jovanović et al., 2014).

**Figure 8:**
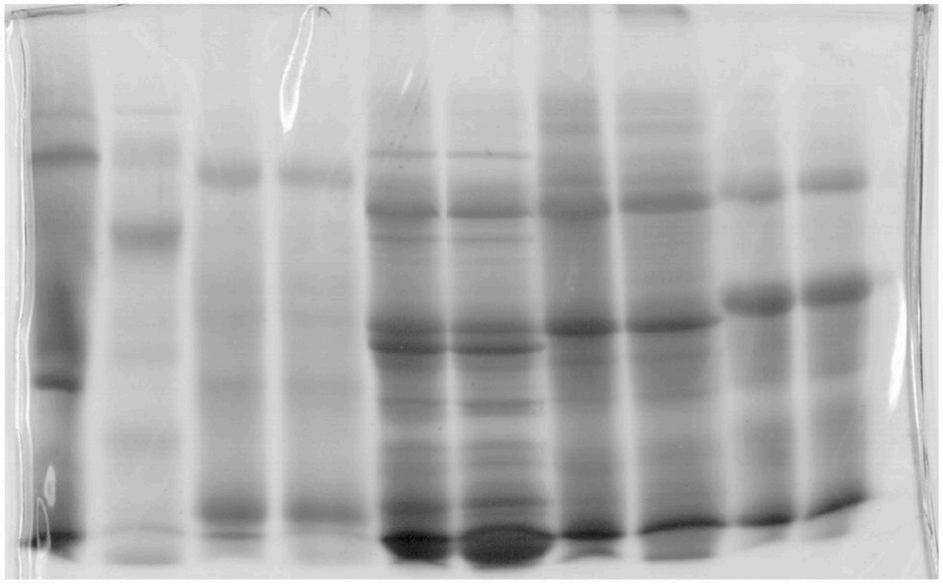
Gel used in molecular biology (Jovanović, 2001; Jovanović et al., 2014).

**Figure 9:**
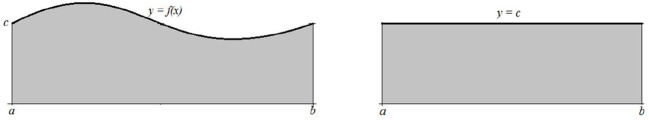
Integral computation.

In last few decades we are witnessing impressive developments of technologies and methods implemented in biological signals. More powerful instrument perception is progressing together with more powerful and more sophisticated methods.

## Basics

A biological signal, coded in computer as a digital function, is usually a finite approximation of an analog signal. Consequently, sampling resolution should be sufficient in order to provide a quality acquisition, enabling detection, extraction, recognition, and normalization of important features in signals and adequate comparison with etalons. Moreover, the successful implementation of mentioned procedures can be further enriched to fully or highly automated systems for classification, reasoning, and decision making. This aim is the essential improvement of the previously achieved insights.

The older (we can say classical) methods, that are usually simple, do not necessarily lead to simplifications, though they are often ballasted with certain semantic limits. On the other hand, the more modern and sophisticated methods do not necessarily improve our knowledge. In the case of careless application, they might lead to false understanding with broader poor consequences. Some of issues related to methods for computation of Granger causality were discussed in (Kasum et al., 2015).

The application of Mathematics and Statistics require permanent criticism and scrutiny, especially in the points where these are connected to non-mathematical semantics. The proper mix of simple and complex modeling could offer substantial advantages.

The initial signal usually requires preprocessing involving different types of normalizations. The standard examples are:

- Filtering of electrophysiological signals;
- Filtering of microscopic optic signals and certain preprocessing operations, e.g., determination of contours of microscopic objects or their nonlinear transformations, or determination of contours of spectrogram features.

Discrete and continual counting measures normalized to the real unit interval are the most prominent measures present in expression of the observed statistical dependences, statistical analysis of the experimental data, probabilistic estimations on finite domains, or on more abstract mathematical structures.

Biomedical statistical analysis involves comparison with the control group, computation of the relevant statistics (e.g., mean, variance, correlation coefficient, *p*−value, *F*− statistics etc.), determination of the regression curves (so called curve fitting) and so on. Statistics has proved to be a very important and powerful mathematical method in biological experimentation, indispensable in the contemporary research and scientific publishing. Conceptual correspondence between the initial measurement and their statistical interpretation always require additional observation and unbiased critical analysis of the initially proposed connections between the natural semantics of the studied phenomena and the constructed mathematical (statistical) model. The following examples will illustrate main issues with semantic stability in the above connection. For basic concepts about statistics we refer the reader to the textbook (Spiegel and Stephens, 2018).

**Example 2.1** A simple sinusoidal oscillation *y* = *f*(*x*) around *c* on the interval [*a, b*] will be by the application of the standard signal energy measure $\overset{b}{\underset{a}{\int }}f\left(x\right)dx$ identified with its mean value, which is the constant function *y* = *c* (Figure 6.)

As a consequence of the integration, any additional information that the initial signal carry will be lost. □

**Figure 10:**
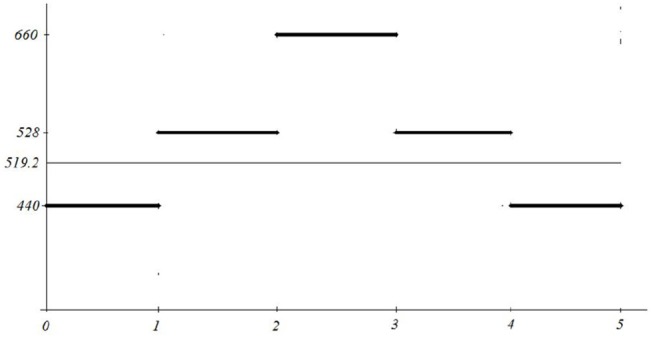
Simple calculation of the mean and its semantics.

**Example 2.2** Consider the signal *y* = *f*(*x*) defined by

$$\begin{array}{l} f\left(x\right)=440\cdot 1_{\left[0,1\right)}\left(x\right)+528\cdot 1_{\left[1,2\right)}\left(x\right)+660\cdot 1_{\left[2,3\right)}\left(x\right)\\ \;+528\cdot 1_{\left[3,4\right)}\left(x\right)+440\cdot 1_{\left[4,5\right]}\left(x\right) \end{array}$$

for 0 ≤ *x* ≤ 5. Its average energy $\overset{5}{\underset{0}{\int }}f\left(x\right)dx$ is adequately represented by its mean value *y* = 519.2 (see Figure 10).

However, the above signal can be interpreted as a producing the equal length tunes *a, c*_1_, *e*_1_, *c*_1_, *a* (frequencies on the *y* axis, A-minor chord), while the corresponding mean 519.2, is atonal and does not have any meaningful connection with the played melody.

## Method

### Complexity Issues

While dealing with simple signals, with simple changes in time, the direct simple representation/visualization is often satisfactory. However, the study of subtler details and processes, and integration of system insight, requires increased complexity. The modern research demands, with invisible important features, higher complexity in representations, and involved structures. This is the point of departure from the simple and simplest representations and measurements, thus opening room for more complex functions and structures and consequently, for more complex measures and operations on these structures. It is very difficult to determine what would be the upper bound for complexity of mathematical structures when dealing with biological signals. Especially now when everybody is aware that neurological signals are directly related to the processing of sensory information and system control in all variety of situations. As an illustration, we refer the reader to the concept of Granger causality that has been extensively used in neuroscience, see (Granger, 1969, 1980; Granger and Morris, 1976; Geweke, 1982, 1984; Kaminski and Blinowska, 1991; Sameshima and Baccala, 1999; Baccalá and Sameshima, 2001a; Baccala and Sameshima, 2001b; Kaminski et al., 2001; Brovelli et al., 2004; Babiloni et al., 2005, 2007; Wang et al., 2007; Blinowska, 2008, 2011; Kuś et al., 2008; Takahashi et al., 2008; Blinowska et al., 2010; Brzezicka et al., 2011; Kasum et al., 2015).

Moreover, and much more important, biological signals like DNA sequences are information bearing structures (even more, they are knowledge bases) and should be treated as such. The particular DNA molecule should be also studied by its set of consequences, not solely by its morphological properties. It seems prudent to involve the entire data science and a significant part of mathematical logic into foundations of biology.

For example, propositions “today is Tuesday” and “it is not true that today is not Tuesday” have the same meaning, but syntactically are quite different. In terms of Euclidean metrics (the main tool for the similarity estimation), they are quite distant. Thus, the syntactical similarity can be quite different form the more important, semantic similarity. The syntax similarity only works properly if applied on objects in normal form (a concept similar to disjunctive or conjunctive normal form in propositional logic).

Back to DNA, we may ask the following questions:

- Is there a normal form of a DNA sequence?
- If the answer is positive, are the DNA molecules always in the normal form?
- What are the properties of the “gene to protein” relation?
- Can we produce an axiom system and derivation rules (i.e., logic) for the synthesis of proteins?

It is not our attention to dispute the well-established use of the Hilbert space formalism in acquisition of biological signals. However, it cannot be the sole mathematical apparatus used in biology, since it offers nothing about consequence relations and deduction in general. It seems prudent to involve some other mathematical disciplines related to automated reasoning. For the reader unfamiliar with the basic concepts of mathematical logic we refer to (Mendelson, 1997).

This is why more complex methods are finding applications and are well emancipated in the processing of biological signals. Here we shortly summarize some elements with their relevant properties that are already in broader use.

### Measures and Metrics

Where there are measurements, immediately there are measures. The signal processing techniques involve application of different kind of measures: counting cardinality, probabilistic, vector valued (non-monotonic), common Euclidean geometry measures, special probabilistic Boolean ({0, 1}-valued) filters (those emerge in situations when deciding if an object has certain property or not) and so on. Usually, the sets occurring in experiments are fairly simple in the sense that they can be adequately approximated by finite sets, or by finite Boolean combinations of intervals and points. As such, they can be rather directly and easily measured. Original entities/objects are corresponded to their mathematical representations. Then obviously, a question arises: to what extent are the representations of a certain kind of entities similar/identical, which we resolve obviously with certain distance measurements-metrics between individual representations. Thus representations, no matter how simple or complex, become points in the space of representations and distance measurements directly determine similarity of originals.

However, one should always be aware of the underlying measure algebra, particularly when dealing with probability measures. The main cause of so called probability paradoxes is absence of the precise determination of the underlying measure algebra, i.e., the absence of the precise definition of the set of events that can be measured with the given probability function. For readers unfamiliar with the basic probabilistic concepts we refer textbooks (Attenborough, 2003; Spiegel and Stephens, 2018).

One of the subjects of the contemporary research is the study of the impact of quantum phenomena on complex biological formations, starting from large molecules, to large systems like brain and related biological phenomena e.g., consciousness. Along this line has emerged the awareness of the necessity of precise description and understanding of signals that are more complex and structures, which leads to utilization of more complex sets (events) and measures on them.

An example of this kind would be determination of the geometric probability for the set with fractal or rather complex boundary. Fractals have become broadly present in Biology in representation of biological functions and characterization of their complexity. Functions are sets; events in a probability are sets.

Another example of more complex measures involves Boolean measures on the set of natural numbers ℕ induced by nontrivial filters and their total extensions.

The first measurements of the more complex curves and geometric objects were performed with the discovery and application of the infinitesimal calculus. The definite integral $\overset{b}{\underset{a}{\int }}f\left(x\right)dx$ of a positive function *f* on [*a, b*] returns the surface area of the corresponding curved trapezium bounded with lines *y* = 0, *x* = *a*, *x* = *b*, and *y* = *f*(*x*).

Development of calculus has brought the methods for integration of more complex functions, e.g., functions with countably many jump discontinuities and functions with essential discontinuities. The abstract concept of an integral has been finally shaped with Lebesgue's theory of measure and integration.

Starting with the basic geometric measures arising from Euclidean metrics (length of a straight line, area of a rectangle, volume of a cube); the measure of more complex sets is determined by application of the σ−additivity property:

$$\begin{array}{l} \mu \left(\overset{\infty }{\underset{n=0}{⋃}}A_{n}\right)=\overset{\infty }{\underset{n=0}{\sum }}\mu \left(A_{n}\right) \end{array}$$

for pairwise disjoint sets *A*_*n*_, *n*∈ℕ,

For example, let

$$\begin{array}{l} A=\overset{\infty }{\underset{n=0}{⋃}}\left[n,n+\frac{1}{2^{n}}\right]. \end{array}$$

Since

$$\begin{array}{l} \left[n,n+\frac{1}{2^{n}}\right]\cap \left[m,m+\frac{1}{2^{m}}\right]=\emptyset \end{array}$$

for *m*≠*n*, and $\mu \left(\left[n,n+\frac{1}{2^{n}}\right]\right)=\frac{1}{2^{n}}$, we obtain that

$$\begin{array}{l} \mu \left(A\right)=\overset{\infty }{\underset{n=0}{\sum }}\frac{1}{2^{n}}=1. \end{array}$$

*The main feature of the zero*−*measure sets*. Precisely, if *E* is a set of the positive measure, *Z* is a zero−measure set (i.e., μ(*Z*) = 0), then for any Lebesgue integrable function *f*, the following is true:

$$\int_{E}^{\;}f\left(x\right)d\mu =\int_{E\setminus Z}^{\;}f\left(x\right)d\mu$$

This was a significant improvement of the Riemann integral.

The modern understanding of a probability is as a normed measure on a probability space. More precisely, probability space is a triple (Ω,A,P), where Ω is a nonempty set of elementary events, A is a σ−complete subalgebra of the powerset algebra P(Ω), and P:A→[0,1] is a σ−additive function such that *P*(Ω) = 1. In particular, P is total if A= P(Ω). These notions can be similarly defined for any other kind of measure.

The additivity *add*(μ) *of* a measure μ is defined as the smallest cardinal number κ such that there is a family *Z* = {*Z*_*i*_:*i*∈κ} satisfying the following two properties:

- μ(*Z*_*i*_) = 0 for all *i*∈κ;
- $\mu \left({⋃}_{i\in \kappa }^{\;}Z_{i}\right)>0.$

Some examples:

1. Calculating area of the curved trapezium;
2. Calculating area of the figure whose boundary has finitely many stepped discontinuities;
3. Calculating area of the figure whose boundary has countably many stepped discontinuities;
4. Calculating geometric probability of the set with simple boundary;
5. Calculating geometric probability of the set with fractal boundary (e.g., Weierstrass function).

Note that *add*(μ) = ω means that μ is *k*−additive for all finite *k*. Measures of various additivity are investigated in theory and used in practice, when additivity can be arbitrary large.

The notions of metrics and measure play important part in modeling of similarity. In the study of information bearing structures, most notably formal deductive systems, it is often easier to define measure than metrics. For example, a consistent propositional theory (set of formulas) *T* naturally induces the corresponding binary measure μ_*T*_ on Lindenbaum algebra *LA* by

$$\mu_{T}\left(\left[\phi \right]\right)=\{\begin{array}{c} 1,\;T⊢\phi \\ 0,\;T⊬\phi \end{array}$$

Here * ϕ* is a propositional formula and [*ϕ*] = {ψ : ⊢ ψ ↔ *ϕ*}.

One of the most common ways to generate metrics from a given measure μ is to measure symmetric difference:

$$\begin{array}{l} d\left(A,B\right)=\mu \left(A△B\right). \end{array}$$

The obtained metrics *d* do not satisfy condition *d*(*A, B*) = 0 ⇒ *A* = *B*, so it is a pseudo-metrics.

### Dimension

The most commonly known meaning of the notion of dimension is that it is the cardinal number of any basis of the given vector space. For example, dimension of the Euclidean space ℝ^*n*^ is, as expected, equal to *n*. Recall that two vector spaces over the same field are isomorphic if and only if they have equal dimensions. This theorem establishes dimension of the vector space as its most important characteristics.

Another important concept of dimension is topological dimension. We shall omit a rather cumbersome technical definition, and try to illustrate the concept in the case of charts. A *k*−dimensional chart in ℝ^*n*^ for *k*<*n* is any subset of ℝ^*n*^ of the form

$$\begin{array}{l} M=\{\left(x_{1}\left(t_{1},\ldots ,t_{k}\right),\ldots ,x_{n}\left(t_{1},\ldots ,t_{k}\right)\right)\;:\left(t_{1},\ldots ,t_{k}\right)\\ \;\in I_{1}\times \ldots \times I_{k}\}, \end{array}$$

where each *I*_*i*_ ⊆ℝ is an interval and each *x*_*i*_:*I*_1_ × … × *I*_*k*_ → ℝ is a smooth function. For example, a sphere with radius *R* = 5 and center at origin is represented by the chart

$$\begin{array}{l} S=\{\left(5\cos\left(s\right)\cos\left(t\right),5\sin\left(s\right)\cos\left(t\right),5\sin\left(t\right)\right)\;:0\leq s<2\pi ,\\ \;-\frac{\pi }{2}\leq t\leq \frac{\pi }{2}\}. \end{array}$$

Generally, a plain curve can be intuitively described as the set of the form

s={(x(t),y(t) :t∈I},

where *I* is an interval. However, various conceptual problems emerge when the additional conditions on coordinate functions *x*(*t*) and *y*(*t*) are omitted. This was explicitly shown by Weierstrass, Dirichlet, Peano, Hilbert, Sierpinski, and others, who constructed quite exotic functions. Some of them are continuous and nowhere differentiable (Weierstrass functions, Koch triangle etc.), space filling (Peano curve is a continuous surjection of the segment [0, 1] onto the square [0, 1] × [0, 1]) and so on. The basic concepts of calculus can be found in textbooks (Attenborough, 2003; Werde and Spiegel, 2010; Hsu, 2014; Spiegel and Stephens, 2018).

Note that if *l* is a graph of a Weierstrass function, then, every two points on it are, measured along *l*, at infinite length. The same is true for fractal curves.

Higuchi fractal dimension procedure became popular with the expanding applications on biological, especially neurological signals. It has been used alone or in combination with other signal analysis techniques in the revealing complexity patterns in the single neuron activity as well as in EEG/ECoG signals that originate from complex neuronal networks in different physiological and pathophysiological conditions (Kesić and Spasić, 2016).

**Example 3.3.1** (Higuchi fractal dimension) In (Kesić et al., 2014) authors use Mean of the empirical *HFD* distribution to investigate the changes in the complexity of snail Br neuron activity after the treatment application. *HFD* measure allows fast computational tracking of variations in signals and in this study has been used in combination with the normalized mean of the empirical *HFD* distribution because the signal of Br neuron activity is non-stationary (Figure 3). This study, among other factors, showed that the normalized mean of empirical *HFD* distribution method is a significant mathematical invariant in monitoring the effects of different treatments on modulation of bursting neuronal activity.

**Figure 11:**
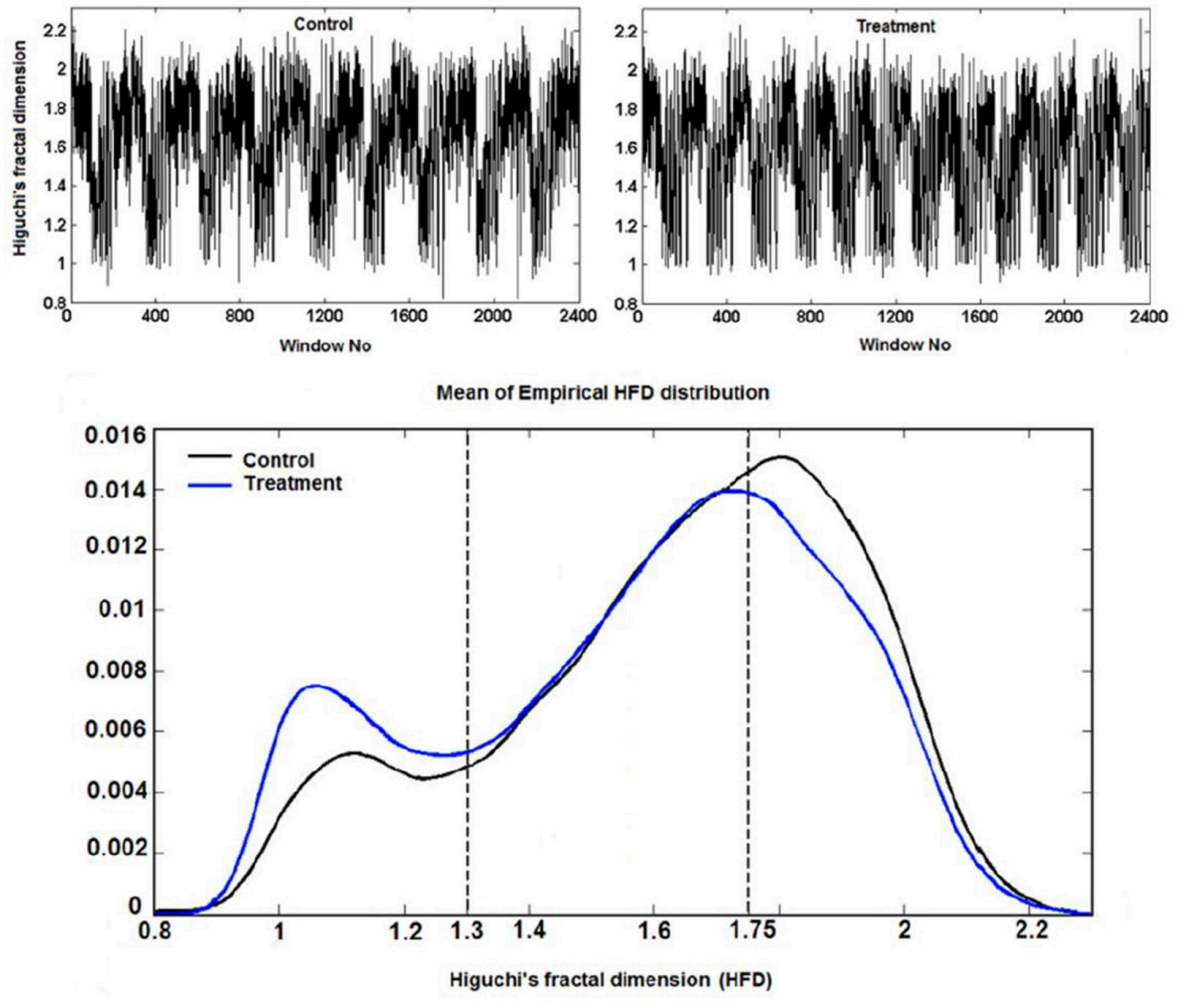
Higuchi's fractal dimension (Kesić et al., 2014).

On Figure 11 is shown typical electrophysiological activity of garden snail Br neuron (60 s) and corresponding Higuchi's fractal dimension (HFD) values in control condition (left column) and after treatment application (right column). Mean of empirical FD distribution of the group in control condition and after treatment application behaves as the mathematical invariant characterizing system modulation.

**Figure 12:**
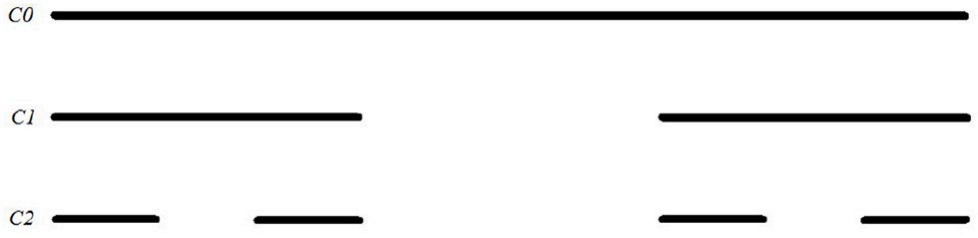
Construction of Cantor set.

In 1918 Felix Hausdorff introduced a generalization of the notion of topological dimension in order to classify objects with fractal boundaries.

**Definition 3.3.2**. (Hausdorff measure) Let (*X, d*) be a metric space and let λ ≥ 0. The Hausdorff measure $H^{\lambda }:P\left(X\right)\to \left[0,+\infty \right]$ is defined by

$$\begin{array}{l} H^{\lambda }\left(S\right)=\underset{\delta >0}{\sup}\inf\left\{\overset{\infty }{\underset{n=0}{\sum }}d{\left(U_{n}\right)}^{\lambda }\;:S\subseteq \overset{\infty }{\underset{n=0}{⋃}}U_{n}\wedge \left(\forall n\in \mathbb{N}\right)d\left(U_{n}\right)<\delta \right\}.\;□ \end{array}$$

The connection between Hausdorff measure and Lebesgue measure is rather strong, as it is stated by the following theorem.

**Theorem 3.3.3** Suppose that *E*⊆ℝ^*n*^ is a Borel set and that μ_*n*_ is the Lebesgue measure on ℝ^*n*^. Then,

$$\begin{array}{l} \mu_{n}\left(E\right)=\frac{\pi^{\frac{n}{2}}}{2^{n}\Gamma \left(\frac{n}{2}+1\right)}H^{n}\left(E\right).\;□ \end{array}$$

Now the Hausdorff dimension is defined by

$$\begin{array}{l} {\text{dim}}_{H}\left(S\right)=\inf\left\{\lambda \geq 0\;:\;H^{\lambda }\left(S\right)=0\right\}. \end{array}$$

A consequence Theorem 3.3.2 is the fact that topological dimension of any smooth manifold *M* is equal to its Hausdorff dimension dim_*H*_(*M*). In particular:

- ${\text{dim}}_{H}\left(D^{n}\right)=n,\text{ where }D^{n}=\left\{\text{x}\in {\mathbb{R}}^{n}\;:\;||\text{x}||\leq 1\right\}$ is the n−dimensional unit ball in ℝ^*n*^;
- ${\text{dim}}_{H}\left({\left[0,1\right]}^{n}\right)=n;$
- ${\text{dim}}_{H}\left(\left\{\text{x}\right\}\right)=0\text{ for any }\text{x}\in {\mathbb{R}}^{n};$
- dim_*H*_([0, 1] × {0}) = 1.

The more interesting examples are related to various fractals.

**Example 3.3.4** (Cantor set) Arguably, the most important fractal construction method was given by Georg Cantor with his iterative construction of the nowhere dense subset of the real unit interval that is equipotent with the unit interval (i.e., has the same cardinal number). In particular, Cantor set *C* is defined by

$$\begin{array}{l} C=\overset{\infty }{\underset{n=1}{⋂}}\overset{3^{n-1}-1}{\underset{k=0}{⋃}}\left(\left[\frac{k}{3^{n-1}},\frac{3k+1}{3^{n}}\right]\cup \left[\frac{3k+2}{3^{n}},\frac{k+1}{3^{n-1}}\right]\right). \end{array}$$

The intuitive definition goes as follows:

- Start with *C*_0_ = [0, 1];
- Remove the middle third from *C*_0_. More precisely, $C_{1}=C_{0}\backslash \left(\frac{1}{3},\frac{2}{3}\right).$ Note that $C_{1}=\left[0,\frac{1}{3}\right]\cup \left[\frac{2}{3},1\right]$;
- Repeat the above procedure on each closed subinterval. For example, $C_{2}=\left[0,\frac{1}{9}\right]\cup \left[\frac{2}{9},\frac{1}{3}\right]\cup \left[\frac{2}{3},\frac{5}{9}\right]\cup \left[\frac{8}{9},1\right]$;
- $$\begin{array}{l} C=\overset{\infty }{\underset{n=0}{⋂}}C_{n}. \end{array}$$

The corresponding Hausdorff dimension of the Cantor set is given by

$$\begin{array}{l} {\text{dim}}_{H}\left(C\right)={\text{log}}_{3}\left(2\right).\;□ \end{array}$$

**Example 3.3.5** (Cantor comb)

The Cantor comb is the set *CC* = *C*×[0, 1]. The corresponding Hausdorf dimension is calculated as follows:

$$\begin{array}{l} {\text{dim}}_{H}\left(CC\right)={\text{dim}}_{H}\left(C\right)+{\text{dim}}_{H}\left(\left[0,1\right]\right)=\log_{3}\left(2\right)+1.\;□ \end{array}$$

**Figure 13:**
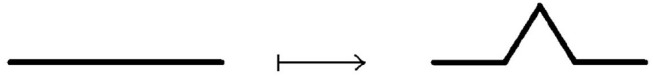
Construction of Koch line.

**Example 3.3.6** (Koch line and Koch snowflake) A Koch line is a planar fractal .whose iterative construction is a modification of the construction of the Cantor set. Instead of deleting the middle segment, over it is constructed equilateral triangle, then its base is removed, as shown on Figure 13.

**Figure 14:**
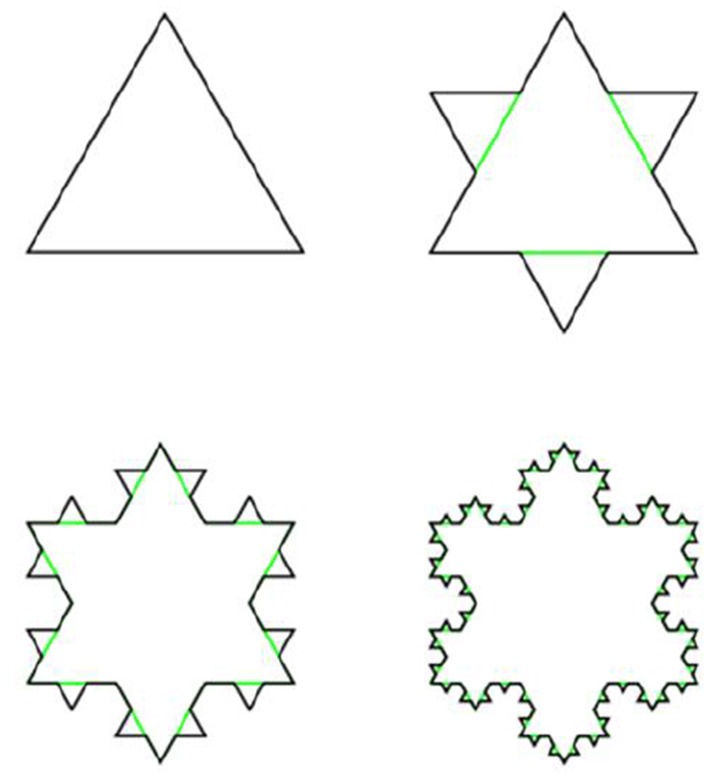
Construction of Koch snowflake.

This procedure is repeated ad infinitum. Similarly, Koch snowflake is constructed from the equilateral triangle by transformation of its edges into Koch lines, as shown on Figure 14.

The Hausdorff dimension of both Koch line and Koch snowflake is equal to log_3_(4).

**Figure 15:**
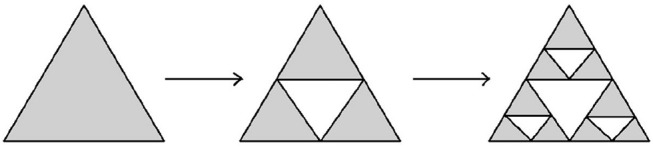
Construction of Sierpinski triangle.

**Figure 16:**
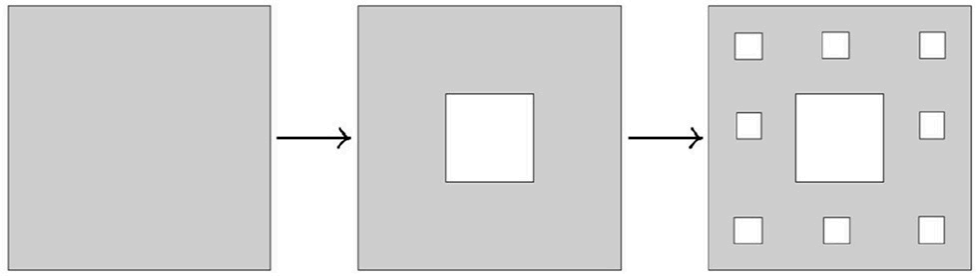
Construction of Sierpinski carpet.

**Example 3.3.7** (Sierpinski triangle and Sierpinski carpet) Another modification of Cantor's iterative fractal construction was introduced by Sierpinski. The corresponding transformations are shown on Figures 15, 16.

The Hausdorff dimension of Sierpinski triangle is equal to log_2_(3), while the Hausdorff dimension of Sierpinski carpet is equal to log_3_(8). □

### Boundary-Interior Index

One of the natural questions involving metric characteristics of a given subset of a metric space is to compare measures of sets and their boundaries. A motivation can be found in classical problems of finding a figure with fixed type of boundary (or fixed measure) with maximal or minimal area or volume. An example of this kind is finding a figure of maximal area whose boundary has the fixed length *l*.

**Definition 3.4.1** Let *A* be a measurable set in ℝ^*n*^. We define the boundary-interior index (BI) of *A* by

$$\text{bi}\left(A\right)=\{\begin{array}{c} \frac{\mu_{n-1}\left(\partial A\right)}{\mu_{n}(A)},\text{ if division is possible}\\ \left(\mu_{n-1}\left(\partial A\right),\mu_{n}(A)\right),\text{ otherwise} \end{array}\;.\;□$$

In the following examples we shall calculate BI for several important sets illustrating characteristic cases.

**Example 3.4.2** (BI of a circle) Let *S* be a circle of radius *R*. Then,

$$\begin{array}{l} \text{bi}\left(S\right)=\frac{\mu_{1}\left(\partial S\right)}{\mu_{2}\left(S\right)}=\frac{2R\pi }{R^{2}\pi }=\frac{2}{R}. \end{array}$$

Moreover, circumference *l* = μ_1_(∂*S*) and radius *R* are connected by the well-known equation *l* = 2*Rπ*, so in terms of circumference *l* we can express the BI of a circle by $\text{bi}\left(S\right)=\frac{4\pi }{l}\;□$

**Example 3.4.3** (BI of *n*−dimensional ball) Let $D_{\text{a},R}^{n}=\left\{\text{x}\in {\mathbb{R}}^{n}\;:\;||\text{a}-\text{x}||\leq R\right\}$ be the *n*−dimensional ball with center **a** and radius *R*. Since $\mu_{n}\left(D_{\text{a},R}^{n}\right)=\frac{{2\pi }^{n/2}}{n\Gamma \left(\frac{n}{2}\right)}R^{n-1}$ and $\mu_{n}\left(\partial D_{\text{a},R}^{n}\right)=\frac{{2\pi }^{n/2}}{\Gamma \left(\frac{n}{2}\right)}R^{n-1},$ it follows that $\text{bi}\left(D_{\text{a},R}^{n}\right)=\frac{n}{R}$. □

**Example 3.4.4** (BI of *n*−dimensional cube) Let *A* = [0, *a*]^*n*^ where *a*>0. Clearly, $\mu_{n}\left(A\right)=a^{n}$. On the other hand, $\mu_{n-1}\left(\partial A\right)=2na^{n-1}$. Thus, $\text{bi}\left(A\right)=\frac{2n}{a}$. □

**Example 3.4.5** Let *H* be the area in Euclidean plane ℝ^2^ bounded with *y* = 0, *x* = 1 and *xy* = 1. Then,

$$\mu_{1}\left(\partial H\right)=\int_{1}^{\infty }\frac{\sqrt{x^{4}+1}}{x^{2}}dx\geq \int_{1}^{\infty }dx=+\infty$$

and

$$\mu_{2}\left(H\right)=\int_{1}^{\infty }\frac{dx}{x}=+\infty .$$

Hence, bi(*H*) = (∞, ∞)

On the other hand, let *T* be the region in Euclidean space ℝ^3^ that is formed by rotation of *H* along the *x* axis. Then,

$$\mu_{2}\left(T\right)=2\pi +2\pi \int_{1}^{\infty }\frac{\sqrt{x^{4}+1}}{x^{3}}dx\geq 2\pi +2\pi \int_{1}^{\infty }\frac{dx}{x}=+\infty ,$$

and

$$\mu_{3}\left(T\right)=\pi \int_{1}^{\infty }\frac{dx}{x^{2}}=\pi .$$

Thus, bi(*T*) = (∞, π). □

**Example 3.4.6** (BI of Koch snowflake *KS*) Let *KS*_*n*_ be the figure obtained in the *n*−th iteration of the construction of the Koch snowflake. Then, $\mu_{1}\left(\partial KS_{n}\right)=3{\left(\frac{4}{3}\right)}^{n}$ and $\mu_{2}\left(KS_{n}\right)=\frac{3\sqrt{3}}{16}{\left(\frac{4}{9}\right)}^{n}$ for *n*>0 and $\mu_{2}\left(KS_{0}\right)=\frac{\sqrt{3}}{4}$, so

$$\begin{array}{l} \mu_{n-1}\left(KS\right)=3\underset{n\to \infty }{\lim}{\left(\frac{4}{3}\right)}^{n}=+\infty \end{array}$$

and

$$\begin{array}{l} \mu_{n}\left(KS\right)=\frac{\sqrt{3}}{4}+\frac{3\sqrt{3}}{16}\overset{\infty }{\underset{n=1}{\sum }}{\left(\frac{4}{9}\right)}^{n}=\frac{2\sqrt{3}}{5}. \end{array}$$

Thus, $\text{bi}\left(KS\right)=\left(\infty ,\frac{2\sqrt{3}}{5}\right)$. If the length of the base of the equilateral triangle *KS*_0_ is equal to *a*>0, then $\text{bi}\left(KS\right)=\left(\infty ,\frac{2a^{2}\sqrt{3}}{5}\right)$. □

**Example 3.4.7** (BI of Sierpinski triangle *ST* and Sierpinski carpet *SC*) Similarly as in the previous example, let *ST*_*n*_ be the figure obtained in the *n*−th iteration of the construction of Sierpinski triangle. It is not difficult to see that $\mu_{1}\left(\partial ST_{n}\right)=3a{\left(\frac{3}{2}\right)}^{n}$ and $\mu_{2}\left(ST_{n}\right)=\frac{a^{2}\sqrt{3}}{4}{\left(\frac{3}{4}\right)}^{n}$, so

$$\begin{array}{l} \mu_{1}\left(ST\right)=\underset{n\to \infty }{\lim}3a{\left(\frac{3}{2}\right)}^{n}=+\infty \end{array}$$

and

$$\begin{array}{l} \mu_{2}\left(ST\right)=\underset{n\to \infty }{\lim}\frac{a^{2}\sqrt{3}}{4}{\left(\frac{3}{4}\right)}^{n}=0. \end{array}$$

Thus, bi(*ST*) = (∞, 0). Here *a* > 0 is the length of the base of the initial equilateral triangle *ST*_0_.

In the case of Sierpinski carpet, $\mu_{2}\left(SC_{n}\right)=a^{2}{\left(\frac{8}{9}\right)}^{n}$ and $\mu_{1}\left(\partial SC_{n}\right)=\frac{a}{2}{\left(\frac{8}{3}\right)}^{n}$, so similarly as in the case of Sierpinski triangle we obtain that bi(*SC*) = (∞, 0) □

**Example 3.4.8** (BI of Cantor comb *CC*) Let *CC* = *C* × [0, 1] be the Cantor comb and let *CC*_*n*_ be the figure obtained in the *n*-th iteration of its construction. Since μ_1_(*C*) = 0 and Fubiuni theorem is true for the Lebesgue integral, μ_2_(*D*) = 0.

On the other hand, $\mu_{1}\left(CC_{n}\right)=2^{n+1}\frac{3^{n}+1}{3^{n}}$, so μ_1_(*CC*) = +∞. It follows that BI has the same value as in the case of Sierpinski triangle and Sierpinski carpet, i.e., bi(*CC*) = (∞, 0). Note that for any *A*⊆ℝ^2^ of positive measure disjoint to *CC*, the set *B* = *A*∪*CC* satisfies condition bi(*B*) = (∞, μ_2_(*A*)).

On the other hand, boundary of Cantor comb contains $2^{{ℵ}_{0}}$ disjoint copies of the segment [0, 1], so, its rectification produces a very long line: a concatenation of $2^{{ℵ}_{0}}$ copies of [0, 1]. Clearly, it cannot be represented by a real number, which opens possibility of facilitating some other types of orderings, e.g., Suslin lines. □

**Example 3.4.9** (BI of Cantor set *C*) It is well known that μ_1_(*C*) = 0. Furthermore, $\mu_{0}\left(C\right)=|C|=2^{{ℵ}_{0}}$, so $\text{bi}\left(C\right)=\left(2^{{ℵ}_{0}},0\right).$ For readers unfamiliar with the basic concepts of set theory we refer to the textbook (Jech, 2006).

The case bi(*A*) = (0, *a*) for *a*∈(0, +∞) is not possible. Indeed, μ _*n*−1_ (∂*A*) = 0 implies that the topological dimension of *A* satisfies condition dim(∂*A*) ≤ *n*−2, hence either interior or exterior of *A* is empty. Consequently, μ_*n*_(*A*) = 0 (case *int*(*A*) = ∅) or μ_*n*_(*A*) = ∞ (case *ext*(*A*) = ∅).

With respect to objects in ℝ^*n*^ for *n*>1 with fractal boundaries, BI behaves like a filter: for fractal ∂*A*, it is either μ_*n*−1_(∂*A*) = 0 or μ_*n*−1_(∂*A*) = ∞.

When there is a need to calculate energy under fractal curve, or further integrate it as with spectrograms, we immediately switch to the 2D or 3D objects with complex-fractal boundary.

### Chromosomes

**Figure 17:**
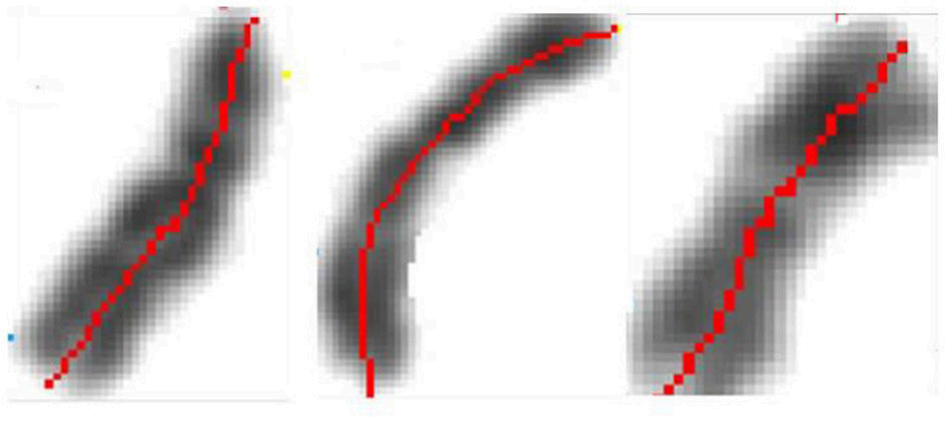
Identification of the central meridian-line of a chromosome before normalization-“rectification,” the feature preparation for the metric-comparison (Jovanović, 2001; Jovanović et al., 2014).

**Figure 18:**
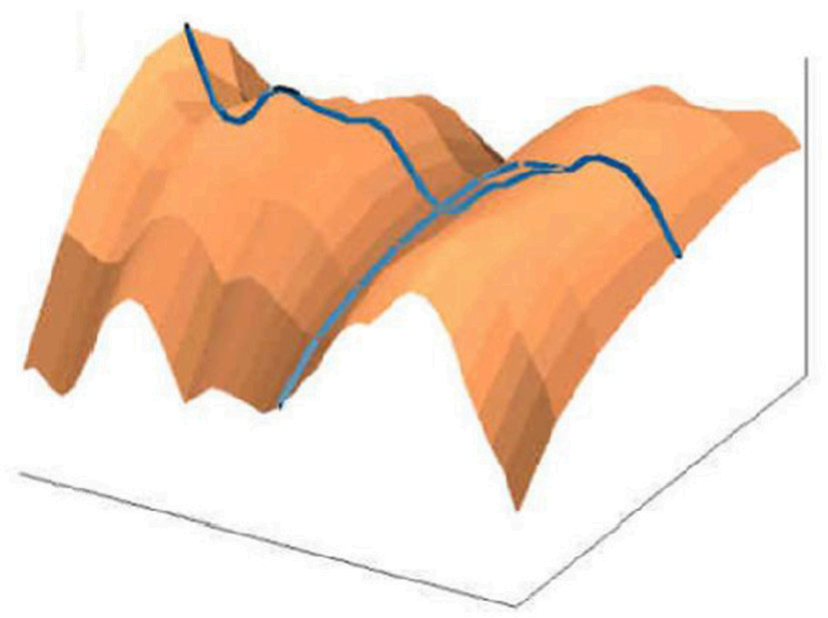
Normalized chromosomal structure detail in chromosomal coordinate system (Jovanović, 2001; Jovanović et al., 2014).

**Figure 19:**
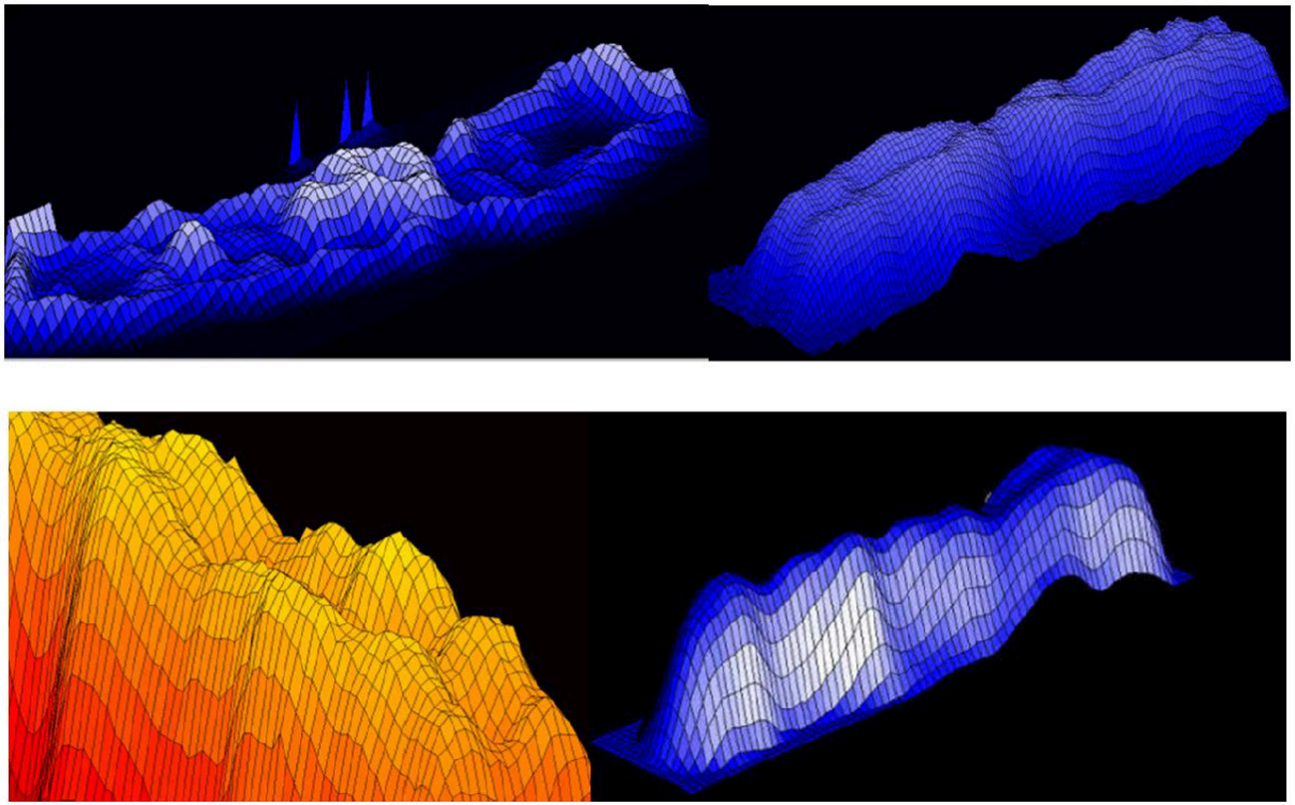
3D representation of absorption in chromosomes; top left, non-normalized –example, the lowest chromosome from Figure 5; the other examples are normalized (Jovanović, 2001; Jovanović et al., 2014).

**Figure 20:**
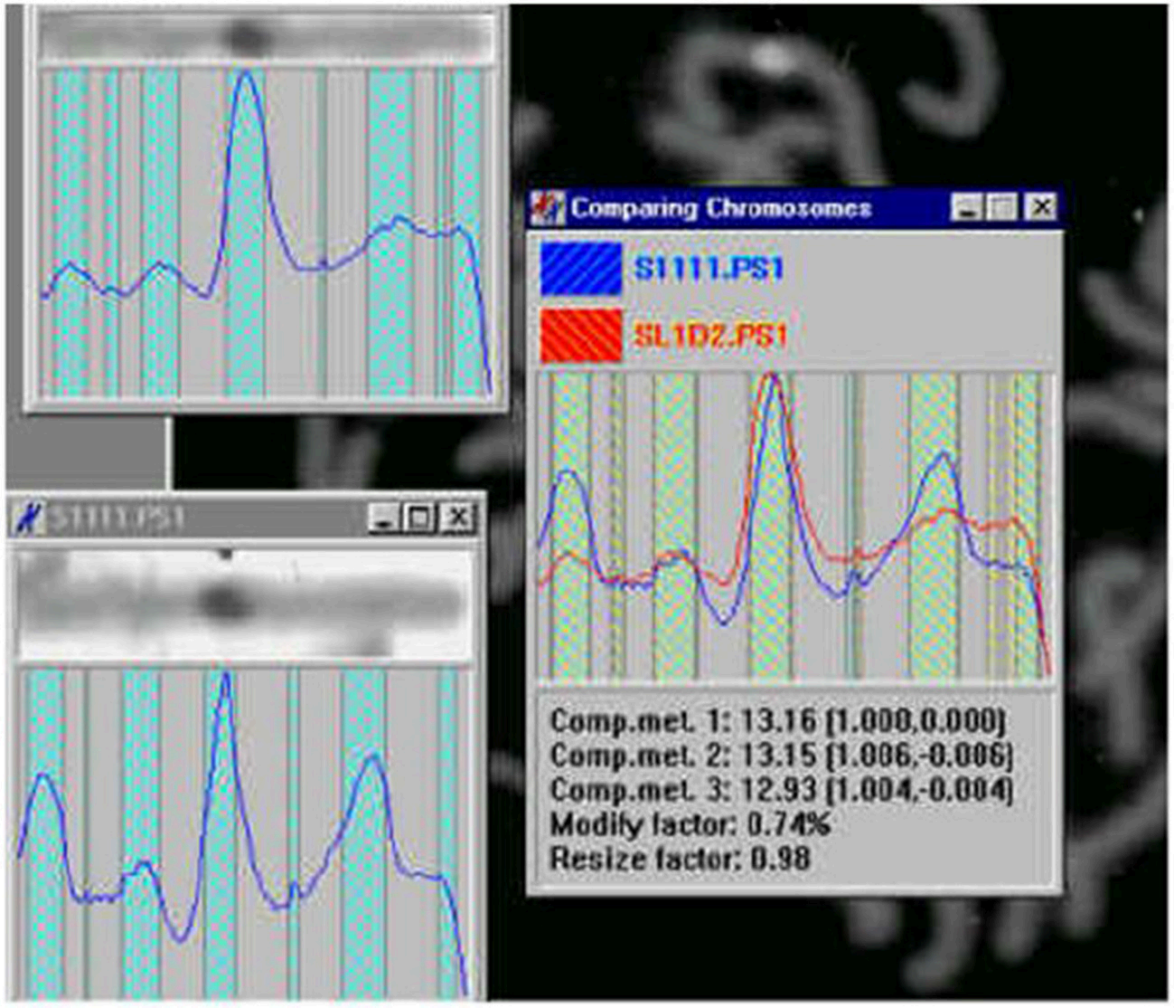
Chromosome measurement, comparison and classification (Jovanović, 2001; Jovanović et al., 2014).

The early image processing initiated the efficient algorithms to penetrate images (Haralick et al., 1973). Extracting objects and operating with them toward desired aims demanded more, e.g., (Vukosavic et al., 2001; Cermak et al., 2016). Every analysis of chromosomes begins with identification of single chromosomes and continues with matching of homolog couples using specific banding patterns, thus reaching the basic karyotyping. The careful visual inspection of small objects in direct observation on photography was the only operation made in genetics for decades. This was seriously extended with CCD microscopy (Jovanović, 2001), when chromosomes became available for mathematical modeling and unprecedented detailed analysis. The gain was lost of subjectivity present in earlier direct visual observations (as illustrated on the Figures 17–20).

**Figure 21:**
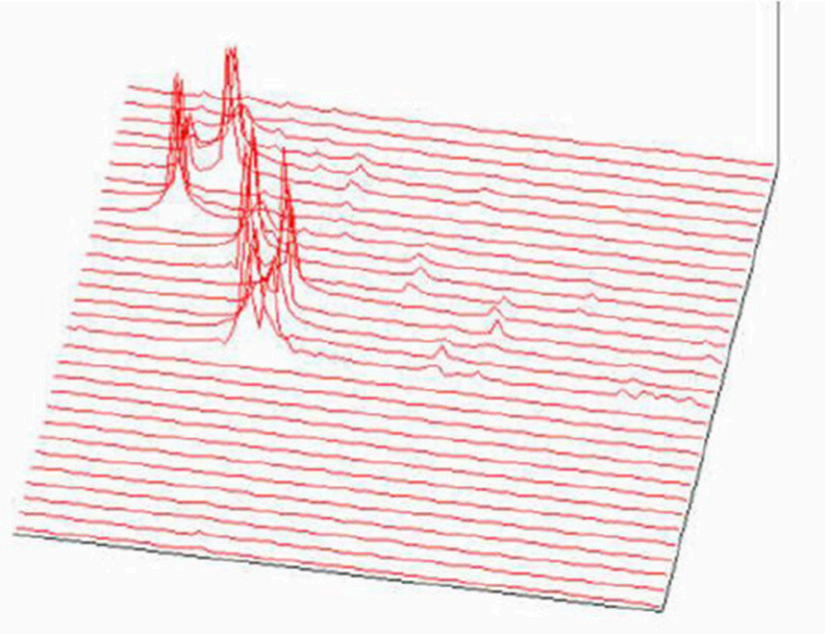
FFT spectrogram as a part of the acoustic melody recognition (Jovanović, 2001; Jovanović et al., 2014).

Genetic content is well ordered within chromosomes, with individual genes located at specific positions, organizing chromosomal coordinate system. Chromosomal (karyotype) classification reached in importance since any change, small or smaller is related to most important life aspects of the studied organism.

The methods and techniques applied in these analyses are expanding at an accelerated rate. Besides karyotyping and its comparisons with the developing standards toward the localization and classification of the individual genes, identification of irregular chromosomes with backtracking of the genetic material forming them, as well as the localization of hardly perceptible (small) fractures and their extraction and further analysis, have been in the research focus (Jovanović et al., 2014).

In the formation of microscopic preparations of chromosomes, they get bent forms randomly. The images (patterns) of light absorption correspond to the absorption intensity two-argument functions are 3D manifolds with characterizing distribution of convex and concave parts (dark and light segments).

The longitudinal distortions bending, unless negligible make direct geometric analysis and comparison hard or non-reasonable.

What initially remains is the investigation of algebraic and topologic invariants of the representing manifolds. Following with multitude of single chromosome shapes we are forced to operate with this representations collected into large sets which is a serious complication. In this preliminary part of chromosomal analysis we recommended a rather simple controlled normalization procedure, as follows (Jovanović et al., 2010).

After the initial contour definition, we form the original chromosomal coordinate system with the orthogonal section lines on the central meridian line. This determines the initial geodesics and the corresponding metrics. By preserving of this central meridian in its original length, using Euclidean distance (which departs substantially from digital-pixel wise distance), rectifying it and positioning the orthogonal lines in the original points, we obtain the receiving Euclidean coordinate network (mesh). This mesh is used to map the original pixels into the receiving orthogonal mesh.

The inflections of the meridian will demand interpolation of pixels in the receiving network, and they correspond to the convex side. The concave-symmetric part will demand pixel fusions in the receiving image, which is the rectified chromosome. Such normalization is very suitable for applications of metrics in order to determine the degree of chromosome similarity with other compared chromosomes, leading rather straight to classification. Thus, rectifying-normalization is intended to produce image of the studied chromosome, as it would be if the chromosome did not have any inflections in the preparation production.

Clearly, smaller inflection enables more precise rectification of the particular chromosome. In cases when the inflection angle induces substantial detail damage, the rectification procedure can be frozen at each desirable angle, thus preserving important image sections, or, extend necessarily the chromosome length.

The alternative procedure is to generate narrow longitudinal bands concentric to the original curved meridian. Those bands should contain the smaller features that are undesirably distorted in the above normalization of the whole chromosome, and rectify only the selected narrow band. This approach will reduce the above disadvantage to negligible.

Once normalized, chromosomal images are well positioned over the simple rectangular domain. Obviously, the algebraic-topological invariants in the original chromosomes are now algebraic-geometric invariants, in the (almost) orthogonal chromosomal coordinate system.

In the early nineties, zooming the chromosome into the chip diagonal, we managed to obtain close to 100 k pixel per chromosome resolution. Now with pixels reduced hundredfold, the number of pixels per chromosome increases proportionally, offering high resolution orthogonal chromosomal systems. The consequence is significant improvement of accessible details within the observed genetic structures. Once when the real chromosome 3D high resolution images become reality, we will deal with the 3D chromosomal orthogonal cylindrical geometry, with appropriate metrics.

In this way, the original chromosome manifold *MCh*_*i*_(*x, y*) converts to the normalized manifold *M*_*i*_(*x, y*). In order to determine chromosomal invariants we determine the following two corresponding sets

$$\begin{array}{l} \bar{M}max_{i}=\left\{\left(x,y\right)\;:\frac{\partial }{\partial x}M_{i}\left(x,y\right)=0\text{ and }M_{i}\left(x,y\right)\text{ is local max}\right\} \end{array}$$

and

$$\begin{array}{l} \bar{M}min_{i}=\left\{\left(x,y\right)\;:\frac{\partial }{\partial x}M_{i}\left(x,y\right)=0\text{ and }M_{i}\left(x,y\right)\text{ is local min}\right\}, \end{array}$$

assuming that the central meridian is collinear with the *x*−axis. The absorption bands tend to form parallel reefs, which will result in certain accumulations along some orthogonal lines on the meridian line. When projected to the meridian (for *y* = 0), they will converge to narrow bands. As representatives of these bands, we take their midpoints and collect them into sets (vectors) *Max*_*i*_.

Similarly form *Min*_*i*_ for the minimums. In this way we can use *Max*_*i*_ as a single simple chromosomal invariant and define measures on such representations which would indicate the level of chromosome similarity and provide general classification. Then for two representation vectors *Max*_*i*_ and *Max*_*j*_ we can define the metrics by

$$d\left(Max_{i},Max_{j}\right)=\sum_{k=1}^{n}\left|x_{i,k}-x_{j,k}\right|{(}^{*})$$

The alternative is to calculate the relative distances of nonzero coordinates of *Max*_*i*_ and *Max*_*j*_ and use these vectors in the metric (^*^). For the alternative purposes we apply more or less refined metrics based on Euclidean metrics, e.g., less refined for global comparisons, more refined for detail inspections.

Earlier we defined some normalized and fuzzy metrics using simplified chromosomal representations. If more detailed and more precise similarity measurement is needed, for the representing set *Max*_*i*_ we can take all local extreme structures, instead of the point-wise projections on the meridian lines (thus, 2D structures).

Other complementary structural study of images of chromosomes is supporting operations on chromosomes with multiple FISH signals, and detection of very small features on chromosomes, see (Jovanović et al., 2014), which would include small structural changes and localization of individual genes in the chromosomal coordinate system.

## Fourier Spectroscopy

Infinite dimensional function spaces, in particular Hilbert spaces, have become a natural mathematical background for signal processing. A Hilbert space *H* is a normed vector space with a scalar product 〈, 〉 which is also complete, i.e., each Cauchy sequence in *H* is convergent.

A countable Fourier basis of *H* is any subset *B* = {**b**_*n*_:*n*∈ℕ} of *H* with the following properties:

- 〈**b**_*n*_, **b**_*n*_〉 = 1 for all *n*∈ℕ;
- 〈**b**_*i*_, **b**_*j*_〉 = 0 for *i*≠*j*;
- $\text{x}=\overset{\infty \;}{\underset{n=0}{\sum }}\langle {\text{b}}_{n},\text{x}\rangle {\text{b}}_{n},\text{ for all }\text{x}\in H$.

The number ${\hat{\text{x}}}_{n}=\langle {\text{b}}_{n},\text{x}\rangle$ is called the *n*-th coordinate of **x** in the basis *B*. Moreover, the first two properties say that *B* is an orthonormal system of vectors, while the last property says that each vector can be expanded in Fourier series. Moreover, coefficients ${\hat{\text{x}}}_{n}$ are uniquely determined in the following sense: $\text{x}=\overset{\infty \;}{\underset{n=0}{\sum }}a_{n}{\text{b}}_{n}\Rightarrow \left(\forall n\in \mathbb{N}\right)a_{n}={\hat{\text{x}}}_{n}$.

A number of semantic distortions and complications occur if the system *B* is not orthonormal, and have serious impact on the validity of the spectroscopic analysis. In (Blinowska, 2008) authors developed a system of base functions that is neither orthogonal, nor normed, unintentionally opening a serious question of the exact meaning of the observed harmonics. More precisely, beside the regular harmonics, such spectroscopy always induces virtual harmonics and do not preserve projections. In particular, projections of certain signal components often do not carry the same meaning as in the regular case, in fact they become meaningless.

Note that Fourier basis can be uncountable. However, the number of the nonzero coordinates is at most countable, which is the statement of the classical theorem that is stated below:

**Theorem 4.1**. Suppose that *B* = {**b**_*i*_ : *i* ∈ *I*} is any Fourier basis of the Hilbert space *H* and that **x** ∈ *H*. Then the set $\left\{i\in I\;:\;{\hat{\text{x}}}_{i}\neq 0\right\}$ is at most countable. □

In signal processing, the standard Hilbert space is the completion of the space of continuous functions on the closed interval [−π, π]. Recall that the scalar product is defined by

$$\langle x,y\rangle =\int_{-\pi }^{\pi }x\left(t\right)y\left(t\right)dt.$$

The corresponding standard Fourier basis *B* is defined by

$$\begin{array}{l} B=\{\frac{1}{\sqrt{2\pi }},\frac{\cos\left(t\right)}{\sqrt{\pi }},\frac{\sin\left(t\right)}{\sqrt{\pi }},\frac{\cos\left(2t\right)}{\sqrt{\pi }},\frac{\sin\left(2t\right)}{\sqrt{\pi }},\\ \;\frac{\cos\left(3t\right)}{\sqrt{\pi }},\frac{\sin\left(3t\right)}{\sqrt{\pi }},\ldots \}. \end{array}$$

Discrete Fourier transform and the fast Fourier transform (FFT) are the most common and most popular methods for the expansion of the numerical vector **x**(*t*) in the standard Fourier basis. Starting from its development in late sixties, it has founded a quite remarkable application in signal processing. In particular, it has enabled discovery of the efficient method for determination of periodic components of the given signal. The usual preprocessing involves various normalization techniques that ensure semantic stability. Readers unfamiliar with the basic concepts of the Fourier spectroscopy we refer to (Attenborough, 2003; Hsu, 2014).

One of the main assumptions is that a given signal **x**(*t*) is stationary, or relative stationary on the observed time interval *L*, i.e., that **x**(*t*) do not have significant changes for *t*∈*L*. It is often a case that authors present and analyze single FFT spectra, without any consideration of signal dynamics. If the signal contains multiple frequency components, say disjoint on the time axis but all within the domain *L* of the performed FFT, then the present frequency components will be detected without time resolution.

Consequently, in order to isolate and extract disjoint periodic component of a signal, it is necessary to successively perform the FFT with a *t* time slide at each iteration and the corresponding shortening of the initial signal. Note that this procedure requires appropriate sample rate and resolution.

For instance, performing FFT for the signal from the Example 2.2 with *L*≥5, we can detect all frequency components 440, 528 and 660, but without time separation, i.e., they will appear simultaneously. However, performing five FFT's with time sliding Δ*t* = 1 five times, each time at the interval [*k*−1, *k*−1+Δ*t*], we shall obtain five spectra, each of them containing exactly one of the frequency components.

Furthermore, reducing *L* to 0.5 and Δ*t* to 0.1 will produce 45 spectra each of them with a single frequency components, or two at the switch zones. In this way, we have approached the criterion of spectral stability and obtained time spectra—a spectrogram with separated tones and melodies within the signal, with the more precise determination of the basic frequencies, intensities, and duration of its components, conditions present in the change of frequency components. This provides a more complete insight into dynamics of a signal.

In particular, with a spectrogram with 50 equidistant spectra we can compensate possibly or certainly erroneous insight and understanding of circumstances induced by analysis of single spectra. Applying some interventions on Fourier spectrograms, e.g., (Jovanović et al., 2010; Spasić et al., 2010), it is possible to achieve very sharp time resolution of the present frequency components and their realistic magnitudes.

**Figure 22:**
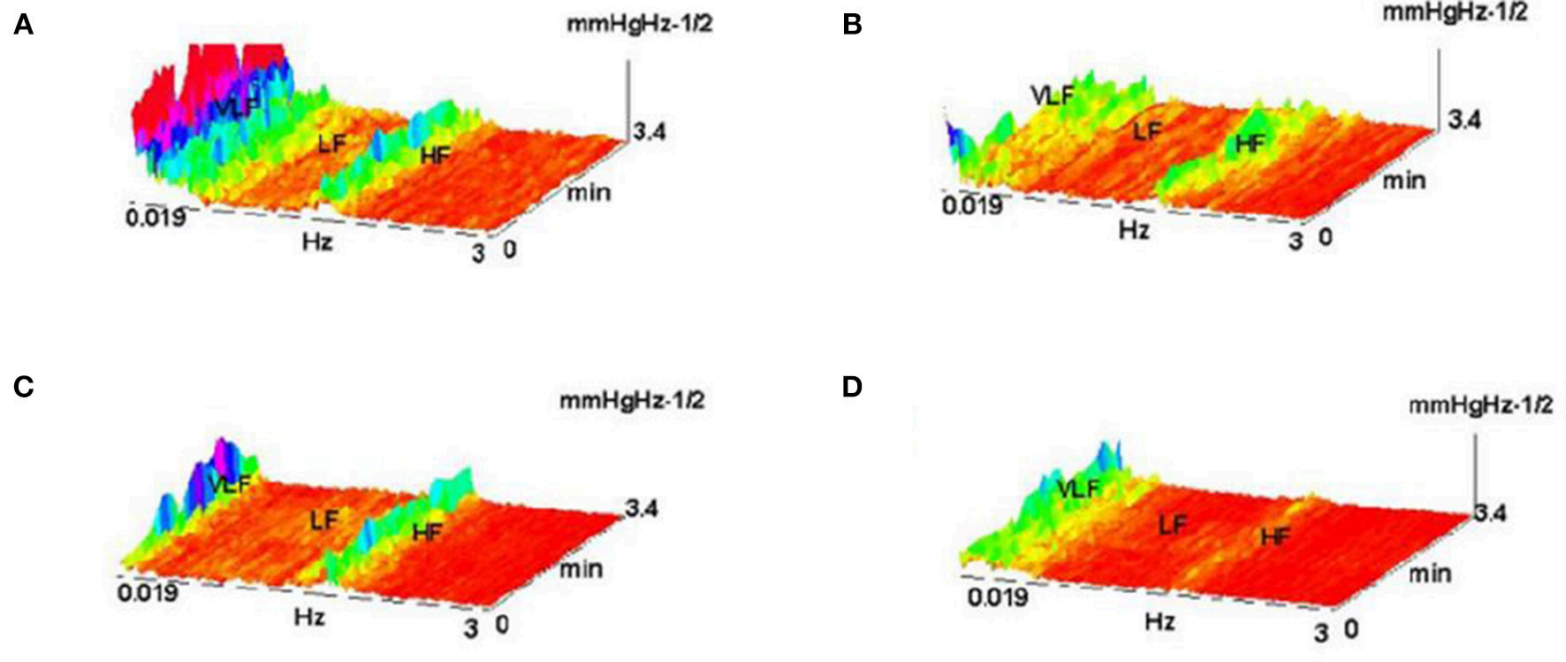
Shown are FFT spectrograms of arterial pressure –AP in hemorrhage experiments, exhibiting the actions of AP modulators present in the AP regulation (system antagonists: renin angiotensin, of sympathetic nervous system and vasopressin). **(A)** hemorrhage 10%; **(B)** hemorrhage 10% pretreated with captopril; **(C)** the same, pretreatment withy phentolamin; **(D)** pretreatment with the substance V2255 (Jovanović, 2001; Jovanović et al., 2014).

**Figure 23:**
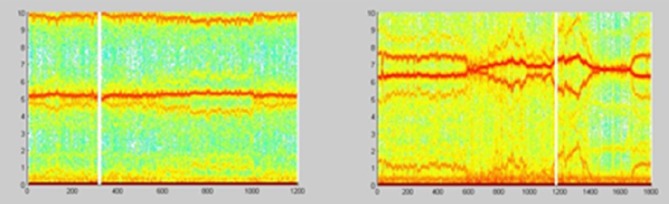
Spectrograms showing the normal AP state and the spectrogram changes and regular feature destructions after administration of scopolamine methyl nitrate (Jovanović, 2001; Jovanović et al., 2014).

The example on Figure 22 (Culić and Šaponjić, 1998; Japundzic-Zigon, 1998, 2001; Japundzic-Zigon et al., 2004) with hemorrhage offers insight into the effects of different substances on AP, the modulators of AP regulating system. Different approaches are available for single sort of experiment. For example presence/absence of major frequency features and their relative relationship, like frequency shifts and power ratios. The low frequency range LF and high frequency-HF, at the middle of frequency interval are essential features in these experimentation, yielding spectrogram characterizations of investigated physiology, which converge to the binary measure form- Y/N, filters, if/when, for example, distinguishing between control, and experimental group, as in the experiment in Figure 23, distinguishing spectral morphology of normal-control state and modulated spectrogram after administration of scopolamine methyl nitrate.

Some other issues are related to semantics of the signal processing by Fourier spectroscopy, see Spasić et al. (2010). Here we shall emphasize the following two important moments. A good time spectrogram can contain features significant for the studied process. Such content often require higher order spectroscopy, with or without the preprocessing involving normalization and application of various measurement, (see Jovanović et al., 2010; Perović et al., 2013).

Secondly, if the spectrogram contains small, hardly detectible, or imperceptible components in some cases they can be detected and extracted by application of the specific methods developed for the image processing. Some of them are applied for the analysis and detection of small features in chromosomes (e.g., Bradski, 1998; Bouguet, 2000; Welch and Bishop, 2004).

We can conclude that measures applied in various classification problems have better semantic correspondence with the reality when used on sufficiently resolute spectrograms or on their features. Furthermore, it is clear that all relevant measures will involve similar invariants-features, with high context dependence.

Specific situations often change the approach for choice of the adequate measure for the complexity of features. In the case of chromosomes, the Euclidean geometry is replaced by the local chromosomal geometry induced by the corresponding geodesics (contours, meridians). In spectroscopy, possible measures will focus on some of the following.

- Position of dominant lines;
- Dispersion;
- Second order FFT performed on extracted features;
- Counting/comparing of picks within certain frequency range with the threshold ε;
- Binary 0−1 measures defined by filters and maximal filters, for example connected to the position of higher harmonics.

Additional treatment of measures on spectra and spectrograms in more general settings is given and discussed in the next section.

## Brain Connectivity Measures

The EEG resolution (the number of electrodes on the scull) has exceeded 2^8^ points more than a decade. Higher density of electrodes-signals for EEG will increase with technology development, and is expected to reach thousands soon.

The relationship of different signals within integrated neurological functions received significant attention in the last few decades. The focus was mainly on the problem of modeling brain connectivity. Developed models have led to the broad range of applications in numerous experimental laboratories, contributing to the rich discourses of fundamental importance in neuroscience.

Clearly, as every processing in the brain involves certain signal processes in the brain, any investigation of neurological signals almost certainly faces the most complex kind of signals. It is also well known that a highly complex system behavior mimics highly chaotic random systems.

For this reason, the successful modeling of stock market trends by Cleave Granger in late sixties and early seventies (Granger, 1969, 1980; Granger and Morris, 1976), soon after found application in the modeling of brain processes, namely brain connectivity.

The initial Granger causality model improved by Geweke which for vector variables has a form

$$\begin{array}{l} \text{x}\left(t\right)=\overset{p}{\underset{j=1}{\sum }}\text{A}\left(j\right)\text{x}\left(t-j\right)+\;\text{E}\left(t\right), \end{array}$$

where **x**(*t*) = (*x*_1_(*t*), …, *x*_*n*_(*t*)) is a vector of variables, **A**(*j*), *j* = 1, …, *p* is a coefficient matrix defining variable contributions at step *t*−*j*, **E**(*t*) are prediction errors. In terms of frequency, Geweke in (Geweke, 1982, 1984) defined the causality model by

$$\begin{array}{l} \text{x}\left(\lambda \right)={\text{A}}^{-1}\left(\lambda \right)\text{E}\left(\lambda \right)=\text{H}\left(\lambda \right)\text{E}\left(\lambda \right), \end{array}$$

where

$$\begin{array}{l} \text{A}\left(\lambda \right)=-\overset{p}{\underset{j=0}{\sum }}\text{A}\left(j\right)e^{-2i\pi \lambda j}, \end{array}$$

**A**(0) = **I** and **H**(λ) is the system transfer matrix. Then he defined the measure of linear causality of two vectors of variables **x** and **y** at frequency λ by

$$\begin{array}{l} f_{\text{y}\to \text{x}}\left(\lambda \right)=\ln\left(\frac{\left|{\text{S}}_{\text{xx}}\left(\lambda \right)\right|}{\left|{\text{H}}_{\text{xx}}\left(\lambda \right){\text{Σ}}_{2}\left(\lambda \right){\text{H}}_{\text{xx}}^{*}\left(\lambda \right)\right|}\right). \end{array}$$

Here ${\text{H}}_{\text{xx}}^{*}\left(\lambda \right)$ is the Hermitian transpose of **H**_**xx**_(λ), | | denotes determinant and **S**_**xx**_(λ) is the upper left block of the spectral density matrix **S**(λ) defined by

$$\begin{array}{lll} \text{S}\left(\lambda \right) & = & \left[\begin{array}{c} {\text{S}}_{\text{xx}}\left(\lambda \right){\text{S}}_{\text{yx}}^{*}\left(\lambda \right)\\ {\text{S}}_{\text{yx}}\left(\lambda \right){\text{S}}_{\text{yy}}\left(\lambda \right) \end{array}\right]=\text{H}\left(\lambda \right){\text{Σ}}_{2}\left(\lambda \right){\text{H}}^{{}^{*}}\left(\lambda \right),\\ \text{H}\left(\lambda \right) & = & \left[\begin{array}{c} {\text{H}}_{\text{xx}}\left(\lambda \right){\text{H}}_{\text{xy}}\left(\lambda \right)\\ {\text{H}}_{\text{yx}}\left(\lambda \right){\text{H}}_{\text{yy}}\left(\lambda \right) \end{array}\right]. \end{array}$$

Finally, **Σ**_2_(λ) is the matrix of error variance.

The idea of Geweke that directed causality between the two nodes *i* and *j* of the graph consisting of precisely located electrodes-signals needs to be analyzed frequency wise was a substantial improvement, which was followed by other brain modeling teams, some of which received major attention and spread broadly.

In the implementations the major connectivity measures are estimating:

- Connectivity between two nodes *i* and *j*;
- Direction of connectivity between *i* and *j*;
- Intensity of connectivity between *i* and *j*. (1)

All of this properties are integrated into a single measure, while generally neglecting the frequency λ at which causality is constructed, replacing it with the maximum over a frequency interval Λ.

Following Geweke, Kaminski, and Blinowska introduced a modification called direct transfer function, defined by

$$DTF_{ij}\left(\lambda \right)=\frac{\left|H_{ij}\left(\lambda \right)\right|}{\sqrt{\sum_{k=1}^{n}{\left|H_{ik}\right|}^{2}}},$$

measuring causality from *j* to *i* at frequency λ. Initially they started with non-normalized form of DTF using the expression of Geweke (Kaminski and Blinowska, 1991; Kaminski et al., 2001).

Sameshima and Bacala proposed somewhat different approach in modifying Geweke measure (Sameshima and Baccala, 1999; Baccalá and Sameshima, 2001a; Baccala and Sameshima, 2001b), with their partial directed coherence, measuring influence of signal at the node *i* to the signal at the node *j* at frequency λ, formally defined by

$$\begin{array}{l} PDC_{ij}\left(\lambda \right)=\pi_{ij}\left(\lambda \right)=\frac{{\text{A}}_{ij}\left(\lambda \right)}{\sqrt{{\text{a}}_{j}^{*}\left(\lambda \right){\text{a}}_{j}\left(\lambda \right)}}. \end{array}$$

Here **A**_*ij*_(λ) is the *i, j*-th entry of **A**(λ), **a**_*j*_(λ) is the *j*-th column of **A**(λ) and ${\text{a}}_{j}^{*}\left(\lambda \right)$ is the Hermitian transpose of **a**_*j*_(λ).

Earlier, they also introduced the direct coherence measure with the intention to estimate direct connectivity between nodes *i* and *j* at frequency λ. It was defined by

$$DC_{ij}\left(\lambda \right)=\frac{\sigma_{jj}H_{ij}\left(\lambda \right)}{\sqrt{\sum_{k=1}^{n}\sigma_{kk}^{2}{\left|H_{ik}\left(\lambda \right)\right|}^{2}}}.$$

More recently Sameshima and Baccala introduced information PDC and DTF (Takahashi et al., 2010), for which they claimed to measure the information flow from the signal at the node *j* to the signal at the node *i* by

$$\begin{array}{l} iPDC_{ij}\left(\lambda \right)={\bar{\text{A}}}_{ij}\left(\lambda \right){\sigma_{ii}^{-1/2}\left({\bar{\text{a}}}_{j}^{*}\left(\lambda \right){\text{Σ}}_{\text{w}}^{-1}{\bar{\text{a}}}_{j}\left(\lambda \right)\right)}^{-1/2} \end{array}$$

and

$$\begin{array}{l} {iDTF}_{ij}\left(\lambda \right)={\bar{\text{H}}}_{ij}\left(\lambda \right){\rho_{jj}^{\frac{1}{2}}\left({\bar{\text{h}}}_{j}^{*}\left(\lambda \right){\text{Σ}}_{\text{w}}^{-1}{\bar{\text{h}}}_{j}\left(\lambda \right)\right)}^{-\frac{1}{2}}. \end{array}$$

Here ${\text{Σ}}_{\text{w}}\text{=}E\left(\text{w}\left(n\right){\text{w}}^{T}\left(n\right)\right)$ is a positive definite covariance matrix of the so called zero mean wide stationary process **w**(*n*), and ρ_*jj*_ is the variance of the so called partialized innovation process ζ_*j*_(*n*) defined by ζ_*j*_(*n*) = *w*_*j*_(*n*)−*E*(*w*_*j*_(*n*)/{*w*_*l*_(*n*) : *l* ≠ *j*}).

Let us mention that numerous experimental teams used the above measures in their discoveries where the above measures reached highest popularity in the formation and formulation of the key conclusions and results, including further modifications (Brovelli et al., 2004; Babiloni et al., 2005, 2007; Schelter et al., 2005; Chen et al., 2006; Singh et al., 2007; Wang et al., 2007).

In (Kasum et al., 2015) we undertook a thorough analysis of these approaches studying all tiny details in the computation and comparison of these measures on the authors data sets, discovering certain inconsistencies and problems involving these measures which substantially compromise their application in some important issues (Kuś et al., 2008; Takahashi et al., 2008; Blinowska et al., 2010; Blinowska, 2011; Brzezicka et al., 2011).

**Figure 24:**
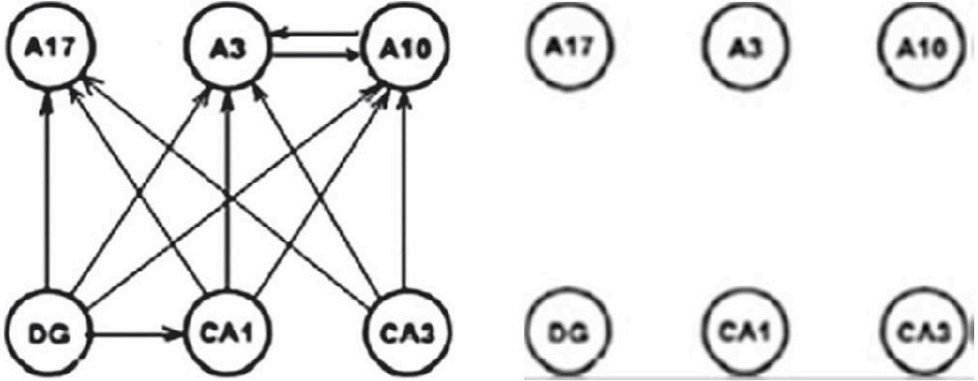
Connectivity difference between DTF and PDC (Kasum et al., 2015).

Presenting three qualities (1) integrally, we are neglecting differences in their importance and masking the most important aspect—being connected. For this reason, we proposed their separated analysis with certain additions, which can result in the different insight of the local inconsistency in the above methods. This is briefly shown on the Figure 24 for a reduced small subset of the system involving 20 × 20 graphs.

On the left diagram is shown connectivity difference between the two measures with corrected statistical zero value. The right diagram contains the same connectivity difference between the two connectivity measures after the natural harmonization of the two experimental zeroes. The consequence is the loss of the connectivity difference in the example illustrating by the authors of PDC the difference and the advantages of their method. Manipulating with different values of statistical zero, one can reach arbitrarily desirable conclusions. Since, we earlier have shown that the DTF is exposing abundant connectivity, when almost everything is connected (D. Adams axiom), now the same will be true for PDC as well, only if sensitivity is sufficiently adjusted, not as far as in the original measure comparisons (Sameshima and Baccala, 1999).

Some alternative approaches were suggested by other research teams (Kroger et al., 2006; Watkins et al., 2006; Jovanović and Perović, 2007; Liu et al., 2007; Aoyama et al., 2009; Klonowski et al., 2009).

On the other hand, we introduced the concept of weak connectivity (Kasum et al., 2015), which might be essential in some processes and might remain hardly noticeable or even imperceptible for the current methods. We offer some alternative methods for detection of such phenomena. One of them is rather simple and goes as follows.

For a set *E* of signals we say that there is connectivity of *E* at frequency λ if

$$\begin{array}{l} {\bar{\pi }}_{E}\left(\lambda \right)=\overset{\;}{\underset{f\in E}{\prod }}PS\left(f\right)\left(\lambda \right)\neq 0. \end{array}$$

Here *PS*(*f*) is the power spectrum of *f*, *PS*(*f*)(λ) is the λ-th coordinate of *PS*(*f*), and ∏ is the coordinate-wise product.

The use of *E* with multiple signals instead of binary *E* might enhance some hardly noticeable periodic components which are present in the whole group of signals. Then, we can say that *E* is connected if ${\bar{\pi }}_{E}\neq \text{0}$, i.e., if ${\bar{\pi }}_{E}\left(\lambda \right)\neq 0$ for some λ.

Besides the above considerations, we also recommended the connectivity being considered over the time interval *T*, rather than at a single point in *T*. The intention is to obtain insights in the time dynamics of connectivity. In this way the usual connectivity directed graphs, after some adaptations, can be substituted with their integration over time. Consequently, this will produce dynamic graph movies instead of single directed graphs, analogous to the spectra—spectrogram integration.

For the power spectra product of the initial time point *t* we can integrate π_*E*_(λ) = π_*E*_(λ, *t*) over *t*∈*T* and λ∈Λ to obtain

$$\begin{array}{l} \pi_{E}\left(\lambda ,T\right)=\overset{\;}{\underset{t\in T}{\int }}\pi_{E}\left(\lambda ,t\right)dt \end{array}$$

and

$$\begin{array}{l} \pi_{E}\left(\lambda ,\Lambda \right)=\overset{\;}{\underset{\lambda \in \Lambda }{\int }}\pi_{E}\left(\lambda ,t\right)d\lambda \end{array}$$

as connectivity measures over the time interval *T* and the frequency interval Λ.

Other methods to establish connectivity on these higher structures are available. Once connectivity between the sets of signals is established, we might consider other two properties: the connectivity direction and intensity.

## Discussion

Biological research, centered on biological signals is in explosive expansion, with neurological contents leading in complexity. With 100 B (Billion) neurons and some its exponent of neuronal connections, the individual brain, as an information processing system responsible for all knowledge accumulated in history, plus a lot of other behavior, exceeds by far the complexity of the whole Internet processing, with all rich parallelism and powerful computational nuclei.

The unknown complexity of individual working brain is far out of reach of our understanding yet. Certainly, it is the most powerful function humanity met in history. Numerous of the processes are multi valued, certain processes binary, dispersed over a range of frequencies. It is the hardest possible approach to learn the unknown functionality from the hardware and individual signal sources. With the simple personal computer it would be a very hard way to reach understanding of software system controls involved, especially all the components of the operating system.

Yet, there are already conferences and discoveries related to the operation of human consciousness, which was until very recently a “nonscientific category.” The approach of parallel investigation of multitude of tasks is promising, as some of the issues are being resolved from multiple projections. The number of combined teams of scientists engaged in brain research is growing, engaging significant resources, which might prove useful.

Mathematical methods briefly discussed here and much more are a product of the brain, thus having its representation and life within the brain much before it is used in brain modeling. Thinking in this way we could be sure all of Mathematics so far applied in biological signals is anything but too complex, as we never experienced the situation when very complex is completely described by very simple.

Nevertheless, we should mention some issues that will be faced sooner in much simpler environments like Quantum Physics and Cosmology. People usually consider Mathematics as a tool set sitting on the shelves, ready to be applied by whomever in whatever capacity and fragments of its developed contents, with all time growing complexity, as natural scientists and engineers are learning more of Mathematics. And this is good, as Mathematics is a public property. The history teaches us that it is hard to guarantee, even for the most abstract parts, that any of discovered Mathematics will never be needed by application. This is the only security for the future of Mathematical funding.

With the growing complexity of the applied mathematical concepts, we are approaching some serious issues of foundations of Mathematics. Before that, let us mention that the symbol ∞ does not represent infinity uniquely since Cantor's discoveries in 1873, when he showed that arithmetical and geometric infinity, i.e., natural numbers and real line are different infinite quantities. As a consequence, infinity has been scaled in terms of pairwise different cardinal numbers. However, the size of this scale is enormous; it cannot be coded by any set. This was the creation of Set theory, and the beginning of the studies of foundations of Mathematics, which is probably never ending.

When dealing with simplest measurements and simplest Euclidean measures we think that everything can be measured. One can only imagine the disappointment of Lebesgue who developed the beautiful completion of measure and integration, when Vitali find a rather simple set on the real line which is not Lebesgue-measurable. In fact there are $2^{{ℵ}_{0}}$ Lebesgue-measurable sets, while there are $2^{2^{{ℵ}_{0}}}$ subsets of ℝ.

The existence of immeasurable sets is highly counterintuitive. These sets cannot be sketched, they are totally amorphous. Sets with fractal boundaries can be seen as a bridge toward the intuitive visualization of immeasurable sets.

From earlier examples, namely, from Lobachevski discovery of non-Euclidean geometries, in twenties of 19th century, which was against all believes of the nature of Geometry, after he showed equiconsistency of the first non-Euclidean Geometry with the anciently perfectly founded Euclidean Geometry which we still learn in the schools, we learned that Mathematical theories, packed around their axioms can be at the same level of logical certainty, while obviously impossible mixed together since with colliding axioms.

And within very short time-a few decades, that discovery gave rise to the huge developments in Geometry, immediately picked up by the most prestigious theoretical physicists as proper Cosmometry (Geometry of the Universe, or its specific parts, e.g., environments of black holes). Concerning the issues related to all measures, we have to say that numerous depend on the axiomatics for Mathematics which is the defining Geometry of the Universe of Mathematics. And there are alternatives combining a smaller set of fundamental axioms and their weaker or stronger versions.

Without entering a discussion that does not belong here, let us just say that AC (Axiom of Choice) is very much needed in the foundations of Mathematics, but there are alternatives. AC implies that Lebesgue measure is not total. However, it implies that there are numerous mentioned measures that are total. Banach proved that there is a total extension of Lebesgue measure which is countably-additive, while, as the Solovay theorem shows (Solovay, 1970, 1971; Pap, 2002), the existence of a total countably-additive extensions of the Lebesgue measure is equconsistent to a very strong property. Some of the functions close to the above-examined fractals are complex enough to open the fundamental issues (for a survey on recent developments in Measure theory see e.g., Pap, 2002).

On the other hand, we can stay on the flat Earth and deal only with short approximation of the phenomena, avoiding entering the zone of the complex Mathematics and its fundamental issues. Yet, as proved by Goedel, we cannot escape the hot issues even remaining only in Arithmetic, nor in any theory containing its copy (like Geometry).

### Other Approaches

Our aim was not to deliver a comprehensive overview of the all metrics and measurements involved in the contemporary biological studies. We have been focused primarily on our work. However, it is prudent to at least mention some of the important topics that are missed here.

The first is related to methods for fractal analysis developed initially for the fractal dimension of observed time series from human physiology and performance. We refer the reader to (Holden et al., 2013).

The second is related to measurement of self-affine structures and a spectrum of scaling parameters. An example of this kind is the detrended fluctuation analysis presented in (Kantelhardt et al., 2002).

The third is related to the recurrence quantification analysis based on the Taken's theorem. For more information we refer the reader to (Webber and Marwan, 2015).

The fourth is related to properties such as ergodicity, anomalous diffusion and multiplicative interactions presented in (Molenaar, 2004; Hasselman, 2013).

The fifth and the final is related to application of non-commutative probabilities presented in (Brovelli et al., 2004; Busemeyer and Bruza, 2012).

## Author Contributions

OK has written the initial draft of sections Measures and Metrics, Dimension, and Boundary-interior index; AJ has written the initial draft of sections Basics, Complexity Issues, Chromosomes, and Fourier spectroscopy (together with AP). AJ and AP have written the initial drafts of Introduction and Discussion. All authors have participated in revision and proofreading of the present version of the manuscript.

### Conflict of interest statement

The authors declare that the research was conducted in the absence of any commercial or financial relationships that could be construed as a potential conflict of interest. The handling editor declared a shared affiliation, though no other collaboration, with the authors at time of review. The reviewer ZMN declared a shared affiliation, with no collaboration, with the authors to the handling editor at time of review.
